# Describing novel mitochondrial genomes of Antarctic amphipods

**DOI:** 10.1080/23802359.2022.2073837

**Published:** 2022-05-10

**Authors:** Louraine Salabao, Tim Plevoets, Bruno Frédérich, Gilles Lepoint, Marc Kochzius, Isa Schön

**Affiliations:** a Laboratory of Functional and Evolutionary Morphology, FOCUS, University of Liège, Liège, Belgium; b Centre for Environmental Sciences, Zoology: Toxicology and Biodiversity, Diepenbeek, Belgium; c Unit Animal Sciences - ILVO Marine Research, Flanders Research Institute for Agriculture, Fisheries and Food, Oostende, Belgium; d Laboratory of Trophic and Isotopes Ecology, FOCUS, University of Liège, Liège, Belgium; e Marine Biology, Vrije Universiteit Brussel (VUB), Brussels, Belgium; f OD Nature, Freshwater Biology, Royal Belgian Institute of Natural Sciences, Brussels, Belgium

**Keywords:** Amphipoda, *Eusirus giganteus*, *Charcotia amundseni*, gene rearrangements, mitochondrial genome, nucleotide diversity

## Abstract

To date, only one mitogenome from an Antarctic amphipod has been published. Here, novel complete mitochondrial genomes (mitogenomes) of two morphospecies are assembled, namely, *Charcotia amundseni* and *Eusirus giganteus*. For the latter species, we have assembled two mitogenomes from different genetic clades of this species. The lengths of *Eusirus* and *Charcotia* mitogenomes range from 15,534 to 15,619 base pairs and their mitogenomes are composed of 13 protein coding genes, 22 transfer RNAs, 2 ribosomal RNAs, and 1 putative control region CR. Some tRNAs display aberrant structures suggesting that minimalization is also ongoing in amphipod mitogenomes. The novel mitogenomes of the two Antarctic species have features distinguishing them from other amphipod mitogenomes such as a lower AT-richness in the whole mitogenomes and a negative GC- skew in both strands of protein coding genes. The genetically most variable mitochondrial regions of amphipods are *nad6* and *atp8*, while *cox1* shows low nucleotide diversity among closely and more distantly related species. In comparison to the pancrustacean mitochondrial ground pattern, *E. giganteus* shows a translocation of the *nad1* gene, while *cytb* and *nad6* genes are translocated in *C. amundseni*. Phylogenetic analysis based on mitogenomes illustrates that *Eusirus* and *Charcotia* cluster together with other species belonging to the same amphipod superfamilies. In the absence of reference nuclear genomes, mitogenomes can be useful to develop markers for studying population genetics or evolutionary relationships at higher taxonomic levels.

## Introduction

Mitogenome DNA sequence data or parts of mitogenomes have been widely used to reconstruct evolutionary relationships or detect cryptic diversity (Caterino et al. 2000; Tang et al. 2020). For instance, in amphipods, sequencing mitochondrial *cox1* or *cytb* together with nuclear genes (e.g. *18S, 28S, ITS2*) has revealed cryptic species of *Hyalella* S.I. Smith, 1874 (Witt et al. 2006), *Caprella penantis* Leach, 1814 (Pilar Cabezas et al. 2013), *Gammarus fossarum* Koch, 1836 (Grabowski et al. 2017) and some *Eusirus* Krøyer, 1845 species (Baird et al. 2011). Molecular data from 13 protein coding genes of *Alicella gigantea* Chevreux, 1899 (Li et al. 2019b), Baikalian amphipods (Romanova et al. 2016), *Gammarus roeselii* Gervais, 1835 (Cormier et al. 2018), *Halice* sp. Boeck, 1871 (Li et al. 2019a), Metacrangonyctidae Boutin & Messouli, 1988 (Bauzà-Ribot et al. 2012), and from all mitochondrial genes (protein coding genes, rRNA, tRNA) of *Gammarus pisinnus* Hou, Li & Li, 2014 and *Gammarus lacustris* G.O. Sars, 1863 (Sun et al. 2020) have been used to reconstruct evolutionary relationships. The broad application of molecular data from mitogenomes can be explained by several advantages, which the mitogenome has compared to the nuclear genome. These include its simpler structure, conserved gene content and limited size (Boore 1999; Li et al. 2019b; Krebes and Bastrop 2012) facilitating sequencing of mitogenomes from those species for which reference nuclear genomes are not yet available. The uniparental, usually maternal inheritance of mitogenomes furthermore simplifies analyses because recombination is either totally absent or very rare (Barr et al. 2005, Lin and Danforth 2004). The relatively high evolutionary rate of mitogenomes generating relative large genetic differences makes mitogenomic DNA sequence data furthermore suitable for studies at the genus or species level investigating population genetic or and phylogeographic patterns (Ballard and Whitlock 2004; Krebes and Bastrop 2012, Tang et al. 2020; Li et al. 2019a). The inclusion of whole mitogenomes has resulted in phylogenies with better statistical supports (Haran et al. 2013; López-López and Vogler 2017) and clearer phylogeographic patterns (Keis et al. 2013). Moreover, despite the highly conserved gene content of the mitogenome, gene order has been found to be variable and can provide additional data for reconstructing phylogenetic relationships and evolutionary histories (Cormier et al. 2018; Krebes and Bastrop 2012; Zhang et al. 2020).

Amphipods are widely distributed crustaceans inhabiting a range of different habitats (Väinölä et al. 2008; Li et al. 2019b). In Antarctica, amphipods are among the most diverse components of the benthic community (Gallardo 1987) and show high levels of endemism (Knox and Lowry 1977) making them ideal model organisms to study evolutionary patterns and divergences based on mitogenomes. Currently, there is only one published complete mitogenome of an Antarctic amphipod, namely of *Gondogeneia antarctica* Chevreux, 1905 (Shin et al. 2012), and no mitogenomes are yet available for abundant amphipods of the genera *Eusirus* Krøyer, 1845 and *Charcotia* Chevreux, 1905.

In this paper, we have assembled and analyzed complete mitogenomes of three Antarctic amphipods from two morphospecies (*Charcotia amundseni* d'Udekem d'Acoz, Schön & Robert, 2018 and *Eusirus giganteus* Andres et al., 2002) and two genetic clades of the latter species. Our aims are to (1) provide full mitogenomic data of selected amphipod species for future research and (2) compare gene content and order with published amphipod mitogenomes to unravel shared and unique patterns of mitogenome evolution in amphipods.

## Materials and methods

### Sample collection

Specimens of two species of Antarctic amphipods, *Charcotia amundseni* d'Udekem d'Acoz, Schön & Robert, 2018 and two genetic clades of *Eusirus giganteus* Andres et al., 2002 (G1 and G2; which might resemble different genetic species (Verheye and D’Udekem D’Acoz 2021) have been collected during different Antarctic expeditions (Table 1) and are curated in the collections of the Royal Belgian Institute of Natural Sciences, Brussels, Belgium.

**Table 1.** Sampling details of specimens analyzed in this study, including date of sample, expedition, locality, geographical coordinates, voucher ID provided by Royal Belgian Institute of Natural Sciences (RBINS), and gear used during sampling.

Species	Date collected	Expedition	Locality, coordinates	Voucher ID	Gear
*Eusirus* cf. *giganteus (G1)*	23 February 2013	PS81, ANT-XXIX-3	Bransfield Strait, 62°43.73′S 57°29.04′W	INV. 122797 spec. C	Agassiz trawl
*Eusirus* cf. *giganteus (G2)*	15 January 2008	CEAMARC	Adélie Coast, 66°10′14.3′S 139°21′11.3″E	MNHN-IU-2019-3365	Beam trawl
*Charcotia amundseni*	23 December 2008	BELARE 08-09	Crown Bay, 70°S 23°E	INV.180000	Baited trap

*Eusirus* cf. *giganteus* (G1) and *E.* cf. *giganteus* (G2) are taxonomically undescribed putative species that belong to *E. giganteus* complexes as verified genetically by Baird et al. (2011) and Verheye and D’Udekem D’Acoz (2021).

*Eusirus* amphipods belong to the superfamily Eusiroidea Stebbing, 1888. *Eusirus* cf. *giganteus* has previously been confused with *Eusirus perdentatus* Chevreux, 1912 due to small morphological differences (Andres et al. 2002). The genetic study of Baird et al. (2011) reveals cryptic diversity of *Eusirus giganteus* including the so-called clades G1–G4, and the existence of a species complex is supported by Verheye and D’Udekem D’Acoz (2021). The same authors report that potential *Eusirus giganteus* species that still need to be formally described showed at least minor morphological differences and different color morphs but that a thorough morphological analysis of the putative genetic species is still required. Given the possibility of multiple cryptic species, we follow here the suggestion of Greco et al. (2021) to use the name *Eusirus* cf. *giganteus* in our study. Our other target species, *Charcotia amundseni,* belongs to the superfamily Lysianassoidea Dana, 1849. The genus *Charcotia* has formerly been known as *Waldeckia* (Chevreux 1905) but recently has undergone a change in nomenclature (D’Udekem D’Acoz et al. 2018) which we follow here.

### Mitochondrial genome sequencing, assembly, annotation, and analyses

DNA has been extracted from a pleopod of each specimen using the DNeasy *Blood* & *Tissue Kit (Qiagen,* Germany*) for both Eusirus cf. giganteus* clades and the Qiamp DNA Minikit (*Qiagen*, Germany) for *Charcotia amundseni* following the manufacturer’s protocol. DNA concentration and quality have been checked with a Nanodrop ND-1000 Spectrophotometer (ThermoFisher Scientific, USA) and a Qubit 2.0 fluorometer (Life Technologies, USA).

A low coverage skimming sequencing approach has been applied at the Genomics Core at the KU Leuven (Leuven, Belgium) using an Illumina HiSeq2500 sequencing platform in the 2 × 125 bp mode. Samples were indexed separately as unique libraries. Reads have been quality-checked using FASTQC (Andrews 2010) and pre-processed with Geneious Prime 2019 v1.8.0 (https://www.geneious.com) by merging paired reads, removing duplicates and trimming of low-quality ends using the BBDuk trimmer in Geneious with the minimum quality set to 20. These pre-processed reads have then been used for *de novo* assemblies in MITObim v1.9.1 (Hahn et al. 2013) with the MIRA 4.0.2 (Chevreux et al. 1999) assembler with default settings (kmer size = 31) and an iteration limit of 100. The *Onisimus nanseni* G.O. Sars, 1900 mitogenome (GenBank accession number FJ555185.1) which belongs to the same superfamily as *Charcotia* and a partial 16S to COI sequence of *Eusirus perdentatus* have been used as seed references. The longest resulting contigs from the *de novo* assembly have been imported into Geneious and further assembled with the ‘map to reference’ approach with medium–low sensitivity and 50 iterations. Identity of the resulting consensus sequences have been verified with BLAST searches (Altschul et al. 1997). Automatic annotation has subsequently been conducted with the MITOS web server, versions 1 and 2 (Bernt et al. 2013). The identity of the *rrnL* region of both *Eusirus* species has been confirmed by BLAST searches only, since it has not been annotated by MITOS. The resulting annotations have been viewed and gene boundaries manually corrected in Geneious. The boundaries of the 13 protein coding genes and 2 rRNA genes have been identified by comparing alignments of the novel assemblies with mitochondrial genes of other amphipod species. Protein coding genes boundaries have been further corrected by avoiding any overlap with the subsequent tRNA gene and by noticing any partial stop codons (T or TA). Such partial stop codons are atypical features of mitochondrial protein coding genes (Cameron 2014). Transfer RNA (tRNA) genes and their secondary structures have been predicted with MitFI (Jühling et al. 2012) in the MITOS pipeline and further verified with ARWEN 1.2.3 (Laslett and Canbäck 2008). Potential control regions (CRs) have been identified from their typical features such as high AT content, poly-T stretches, and hairpin structures (Zhang and Hewitt 1997).

Gene orders of the novel mitogenome assemblies were compared to the putative pancrustacean ground pattern which is derived from both Crustacea and Hexapoda (often referred to as pancrustacea) as they share the same ground pattern in terms of their mitochondrial gene order (Kilpert and Podsiadlowski 2006; Boore et al. 1998). Possible gene rearrangements have been analyzed with the CREx web service (Bernt et al. 2007). CREx utilizes a strong common interval tree to heuristically deduce the plausible rearrangement scenarios to change one gene order to another (Bernt et al. 2007). AT and GC skew have been calculated using the formulas of Perna and Kocher (1995): AT skew = [A − T]/[A + T] and GC skew = [G − C]/[G + C]. Only other amphipod species with complete and published mitogenomes have been analyzed for their AT and GC skew (Supplementary Table 1). Nucleotide diversity (π) has been computed for each protein coding gene with DnaSP v6.12.03 (Rozas et al. 2017).

To verify the phylogenetic position of the studied species, the three novel and assembled mitogenomes were supplemented with data from other amphipod species for phylogenetic reconstructions. Published amino acid sequences of 13 protein coding genes were obtained from GenBank and aligned separately for each gene using MAFFT v7.0 online (Katoh et al. 2019), together with the amino acid sequences of the current study. The resulting alignments were concatenated with Geneious Prime 2019 v1.8.0 (https://www.geneious.com) and trimmed with Bioedit v7.2.5 (Hall 1999) with additional checking by eye. The MtArt + G + F was chosen as the best fitting model of molecular evolution as identified with ModelGenerator v0.85 (Keane et al. 2006) using four discrete categories for gamma distribution. Phylogenetic analyses based on maximum likelihood methods were carried out using PhyML v3.0 (Guindon and Gascuel 2003) with 1000 bootstrap replications. Bayesian inference was conducted with MrBayes v3.2.7 (Ronquist and Huelsenbeck 2003) with 1 million generations, tree sampling every 1000th generation, and 10% of the initial trees being discarded as burn-in.

## Results

### Mitogenome organization

The total length of the obtained complete mitochondrial genomes of *Eusirus* cf. *giganteus* (G1), *Eusirus* cf. *giganteus* (G2), and *Charcotia amundseni* is 15,558, 15,534, and 15,619 bp, respectively (Genbank accession nos. OK489458, OK489459, OK489457, respectively) which is within the range of complete mitogenomes from other amphipods (13,517–18,424 bp) (Table 2). The three newly assembled mitogenomes are each composed of 13 protein coding genes, 22 tRNAs and 2 rRNAs. For *E.* cf. *giganteus*, 23 genes are encoded on the positive (+) strand and 14 on the negative (–) strand while 17 genes are encoded on the + strand and 20 on the – strand in *C. amundseni* (Supplementary Figure 1a and b, Supplementary Table 2). A putative control region (CR) has also be identified in all three mitogenomes and is located between *trnS2* and *rrnL* in *Eusirus* and between *trnF* and *nad5* in *C. amundseni*. The mitogenome also contains 20 intergenic regions for *E.* cf. *giganteus* and 18 intergenic regions for *C. amundseni*. The whole mitogenomes of the two species show AT-richness of 61.9% for *E.* cf. *giganteus* and 68.7% for *C. amundseni*, respectively, which contributes to the positive AT skew (0.008 to 0.092) and negative GC skew (−0.317 to −0.201) values observed in the three mitogenomes (Table 2). A relatively high AT content is also observed in the complete mitogenomes of other amphipod species varying from 61.09 to 77% (Table 2).

**Table 2.** Gene lengths, AT content, and AT and GC skews of *E.* cf. *giganteus* (G1), *E.* cf. *giganteus* (G2) and *C. amundseni* and 40 other amphipod species with complete, published mitogenomes (for details of the analyzed species, see Supplementary Table 1).

		Whole mitogenome			Protein coding genes			tRNA genes			rRNA genes		
Species	Length	A + T%	AT skew	GC skew	A + T%	AT skew	GC skew	A + T%	AT skew	GC skew	A + T%	AT skew	GC skew
***Eusirus* cf. *giganteus* (G1)**	**15,558**	**61.90**	**0.008**	**−0.201**	**59.60**	**−0.003**	**−0.207**	**66.20**	**0.063**	**−0.096**	**69.70**	**0.020**	**−0.260**
***Eusirus* cf. *giganteus* (G2)**	**15,534**	**61.90**	**0.012**	**−0.201**	**59.60**	**−0.007**	**−0.208**	**66.20**	**0.066**	**−0.098**	**69.70**	**0.021**	**−0.261**
***Charcotia amundseni***	**15,619**	**68.70**	**0.092**	**−0.317**	**67.30**	**0.115**	**−0.312**	**69.90**	**0.058**	**−0.220**	**70.80**	**0.057**	**−0.310**
*Alicella gigantea*	16,851	68.44	0.071	−0.301	66.12	−0.122	0.007	65.52	0.027	0.106	69.59	−0.135	0.335
*Ampithoe lacertosa*	14,607	77.00	0.066	−0.153	72.10	0.071	−0.157	75.55	0.092	−0.071	80.17	0.073	−0.216
*Bahadzia jaraguensis*	14,657	69.67	0.037	−0.431	68.48	0.040	−0.454	71.85	0.018	−0.184	72.43	0.076	−0.477
*Brachyuropus grewingkii*	17,118	62.24	0.003	−0.307	60.20	−0.015	−0.295	65.41	0.025	−0.157	66.36	0.074	−0.383
*Caprella mutica*	15,427	68.00	−0.020	−0.170	67.70	−0.140	−0.110	72.00	0.010	−0.112	72.40	−0.050	−0.170
*Caprella scaura*	15,079	66.43	−0.015	−0.134	64.24	−0.028	−0.136	71.09	0.025	−0.139	71.77	0.024	−0.149
*Epimeria cornigera*	14,391	68.11	0.034	−0.357	66.92	0.039	−0.373	69.72	0.031	−0.140	73.54	0.019	−0.384
*Eulimnogammarus cyaneus*	14,370	67.59	−0.019	−0.251	66.78	−0.040	−0.246	66.69	0.017	−0.132	71.81	0.095	−0.377
*Eulimnogammarus verrucosus*	15,314	68.96	−0.007	−0.238	66.63	−0.023	−0.249	67.42	0.022	−0.090	69.54	0.072	−0.348
*Eulimnogammarus vittatus*	15,534	67.42	−0.014	−0.222	65.59	−0.033	−0.226	67.30	0.013	−0.122	71.30	0.072	−0.341
*Eurythenes magellanicus*	14,988	61.15	0.044	−0.388	59.26	0.040	−0.399	64.70	0.042	−0.202	64.65	0.074	−0.443
*Eurythenes maldoror*	14,976	61.53	0.067	−0.430	59.81	−0.152	−0.065	64.65	0.042	0.077	64.86	−0.073	0.459
*Gammarus duebeni*	15,651	64.00	−0.016	−0.223	61.00	−0.038	−0.229	64.00	0.031	−0.121	65.00	0.037	−0.345
*Gammarus fossarum*	15,989	65.14	0.018	−0.261	62.56	0.011	−0.268	66.28	0.027	−0.175	72.46	0.022	−0.269
*Gammarus lacustris*	15,333	64.30	0.014	−0.263	62.09	−0.027	−0.272	65.20	0.021	−0.132	68.50	0.013	−0.305
*Gammarus pisinnus*	15,907	70.00	−0.068	−0.310	68.01	−0.090	−0.332	68.88	0.023	−0.120	73.55	−0.051	−0.322
*Gammarus roeselii*	16,073	66.80	0.016	−0.259	64.38	0.012	−0.266	65.82	0.048	−0.151	69.93	0.087	−0.362
*Gmelinoides fasciatus*	18,114	65.87	−0.027	−0.223	63.29	−0.020	−0.296	66.47	0.058	−0.137	69.00	0.031	−0.332
*Gondogeneia antarctica*	18,424	70.10	−0.006	−0.290	67.00	−0.016	−0.314	69.65	0.021	−0.116	70.25	−0.007	−0.261
*Grandidierella fasciata*	14,656	67.56	0.058	−0.189	65.32	0.060	−0.179	71.26	0.169	−0.184	74.88	0.070	−0.329
*Grandidierella japonica*	14,930	66.91	0.097	−0.189	64.94	0.098	−0.184	70.73	0.088	−0.161	72.30	0.136	−0.263
*Grandidierella osakaensis*	14,658	70.90	0.037	−0.182	69.30	0.042	−0.172	75.75	0.057	−0.093	75.79	0.029	−0.325
*Grandidierella rubroantennata*	14,469	74.14	0.056	−0.232	73.28	0.059	−0.234	76.25	0.065	−0.102	77.00	0.062	−0.344
*Haploginglymus* sp.	15,000	68.53	0.041	−0.396	66.61	0.037	−0.413	71.06	0.021	−0.212	74.26	0.096	−0.450
*Hyalella azteca*	15,991	61.09	−0.066	0.052	59.59	−0.102	0.086	65.83	0.013	0.049	64.33	0.053	0.041
*Metacrangonyx boveei*	15,012	72.59	−0.009	0.005	70.28	−0.017	0.036	75.58	0.036	0.022	75.27	0.024	−0.252
*Metacrangonyx longipes*	14,113	76.03	−0.020	−0.040	75.33	−0.170	0.080	78.01	0.050	0.180	78.70	0.028	−0.271
*Metacrangonyx nicoleae tamri*	13,517	74.02	−0.049	0.111	73.92	−0.083	0.163	76.75	0.004	0.043	78.25	0.015	−0.139
*Metacrangonyx repens*	14,355	76.88	−0.025	−0.014	76.00	−0.038	0.020	78.91	0.007	−0.007	79.22	0.022	−0.280
*Metacrangonyx spinicaudatus*	15,037	74.79	0.010	−0.139	73.25	0.010	−0.126	78.17	0.031	−0.056	77.42	0.013	−0.352
*Onisimus nanseni*	14,734	70.30	−0.004	−0.198	68.60	−0.011	−0.189	73.07	0.004	−0.112	76.25	0.009	−0.286
*Pallaseopsis kesslerii*	15,759	63.10	0.010	−0.182	61.13	−0.018	−0.184	67.52	0.063	−0.069	64.87	0.071	−0.241
*Platorchestia japonica*	14,780	72.58	0.015	−0.237	70.61	0.002	−0.237	76.68	0.055	−0.131	75.40	0.069	−0.338
*Platorchestia parapacifica*	14,787	74.80	0.011	−0.253	73.18	−0.002	−0.253	76.69	0.035	−0.110	77.19	0.066	−0.330
*Pleonexes koreana*	14,645	73.20	0.071	−0.206	70.20	0.078	−0.221	74.70	0.083	−0.044	79.00	0.072	−0.263
*Pseudocrangonyx daejeonensis*	15,069	68.00	0.003	−0.350	66.31	−0.006	−0.350	69.37	0.038	−0.223	73.27	0.034	−0.441
*Pseudoniphargus daviui*	15,157	68.70	−0.002	−0.314	66.40	−0.024	−0.317	70.40	0.015	−0.168	73.80	0.076	−0.433
*Stygobromus indentatus*	14,638	69.00	0.016	−0.270	67.40	0.007	−0.275	71.60	−0.009	−0.173	74.50	0.081	−0.356
*Stygobromus tenuis potomacus*	14,915	69.00	0.020	−0.275	67.20	0.012	−0.284	71.00	0.008	−0.156	73.20	0.095	−0.383
*Trinorchestia longiramus*	15,401	71.20	0.039	−0.277	68.60	0.030	−0.291	74.00	0.067	−0.112	73.90	0.101	−0.325

Target species of the current study are indicated in bold.

### Gene order and rearrangements

A translocation of *nad1* gene in *E.* cf. *giganteus* is observed while *cytb* and *nad6* are translocated in *C. amundseni* as compared to the pancrustacean ground pattern (Supplementary Figure 2). We furthermore also find shifts in the position of tRNAs and the control region in *E.* cf. *giganteus* and *C. amundseni* as compared to the pancrustacean ground pattern (Supplementary Figure 2). While also *trnG* has been translocated in the three species investigated here, we find other tRNA gene strings consisting of *trnA*, *trnS1*, *trnR*, *trnN*, and *trnE* for *E.* cf. *giganteus* and *trnS1*, *trnN*, *trnE*, and *trnF* for *C. amunseni* (Supplementary Figure 2). Similar with the pancrustacean ground pattern, the *trnV* is located between the *rrnL* and *rrnA* genes in *E.* cf. *giganteus* while *trnC* and *trnV* are inserted between these genes in *C. amundseni* (Supplementary Figure 2).

Results of the CREx analyses indicate that *E.* cf. *giganteus* and *C. amundseni* have undergone multiple transpositions and rearrangements relative to the pancrustacean ground pattern (Supplementary Figure 3b).

### Protein coding genes

The most frequent start codon in *E.* cf. *giganteus* and C*. amundseni* is ATG (Supplementary Table 2). Defining the protein coding gene boundaries following a tRNA results in a few partial or incomplete stop codons (T or TA). The AT content of the protein coding genes of the three amphipod mitogenomes is estimated as 59.6% for *E.* cf. *giganteus* (G1 and G2) and 67.3% for *C. amundseni* (Table 2). Mitochondrial genomes of the two species in this study have negative GC skew values in the protein coding genes encoded on both strands (Supplementary Table 4). The highest AT content is found in the third codon position of *C. amundseni* and the second codon position of *E.* cf. *giganteus* while the lowest AT content is observed in the first codon position in all three species. (Supplementary Table 3).

### Ribosomal RNA

The two ribosomal RNA (*rrnS* and *rrnL*) genes in the three mitogenomes are located on the negative (−) strand. In the two *Eusirus* species, the length of both RNAs is 681 bp and 871 bp, respectively (Supplementary Table 2). Unlike in *E.* cf. *giganteus*, the two mitochondrial rRNAs of *C. amundseni* are shorter (529 bp and 739 bp) (Supplementary Table 2). The mitochondrial rRNA genes of the two amphipod species in this study also show a high AT content with 69.7% for *E.* cf. *giganteus* (G1 and G2) and 70.8% for *C. amundseni*.

### Transfer RNA

In the three mitogenomes of this study, 22 tRNAs are present with a length ranging from 52 to 67 base pairs (Supplementary Table 2). The AT content of tRNAs of *E.* cf. *giganteus* is 66.2% and 69.9% for *C. amundseni* (Table 2). In *E.* cf. *giganteus*, 14 tRNAs are encoded in the + strand and 8 in the − strand. In *C. amundseni* 10 tRNAs are encoded in the + and 12 in the − strand. Typical clover leaf secondary structures are observed in most predicted tRNAs although some tRNAs show wobble base pairs, atypical pairing or the DHU and TΨC arm are missing (Supplementary Figure 4a–c). More specifically, the DHU arm is missing in *trnS1*, *trnS2* and *trnV* of *E.* cf. *giganteus* (Supplementary Figure 4a and b) and *trnS1*, *trnS2*, and *trnI* of *C. amundseni* (Supplementary Figure 4c). We also find that the TΨC loop is absent in *trnK*, *trnD*, *trnN*, *trnM*, *trnS2*, *trnI,* and *trnQ* of *E.* cf. *giganteus* (Supplementary Figure 4a and b) and in *trnD*, *trnH*, *trnL1*, *trnC*, *trnV*, *trnQ*, *trnK*, and *trnM* of *C. amundseni* (Supplementary Figure 4c).

### Nucleotide diversity

When estimating nucleotide diversity (π) between the two *Eusirus* genetic clades, we observe high values for *nad6* (0.013), *nad5* (0.012), and *nad1* (0.011) (Supplementary Figure 5a) and low ones for *nad4* (0.002), *nad3* (0.003), *nad2* (0.004), and *cox1* (0.005) with *nad4* (0.002) being the least variable. We also find high variability in *nad6* (0.569), *atp8* (0.566), and nad2 (0.515) between *C. amundseni* and *E.* cf. *giganteus* (G1) and low variability in *cox1* (0.279), *cytb* (0.328), and *cox3* (0.342) (Supplementary Figure 5b). Moreover, between *C. amundseni* and *E.* cf. *giganteus* (G2), high variability is observed in *nad6* (0.567), *atp8* (0.560), and *nad2* (0.515), while the lowest variability is found in *cox1* (0.28), *cytb* (0.327), and *cox3* (0.343) (Supplementary Figure 5b).

### Phylogenetic analysis

**Figure 1.**
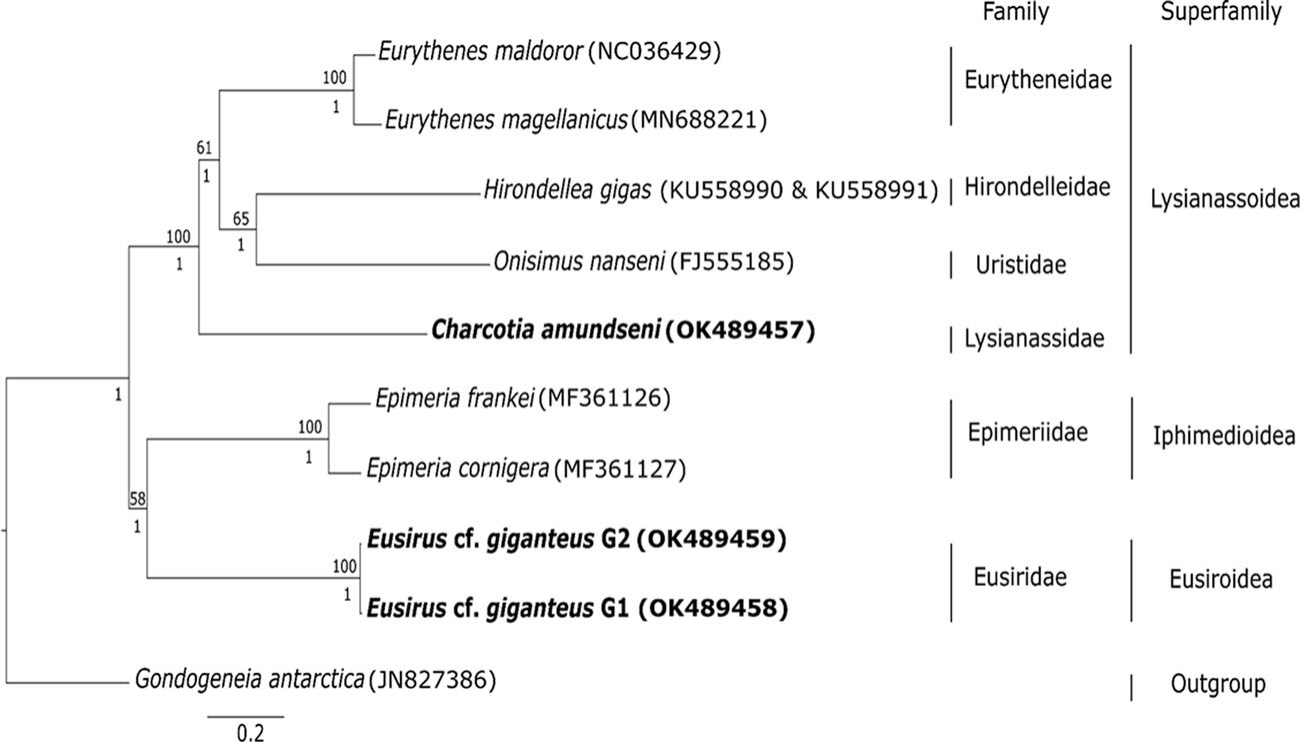
Phylogenetic tree based on the concatenated 13 protein coding genes amino acid alignment using maximum likelihood and Bayesian methods. Only bootstrap values of ≥50 (above the nodes) and posterior probabilities >0.80 (below the nodes) are shown. Scale bar corresponds to the number of substitutions per site. Target species of the current study are indicated in bold. The Genbank accession numbers for the mitochondrial genomes are shown in the parenthesis.

Phylogenetic analysis revealed that the phylogenetic grouping follows the superfamily identity and fall under different family groups (Figure 1). The two genetic clades of *Eusirus* cf. *giganteus* G1 and G2 under family Eusiridae, cluster together. They belong to the superfamily Eusiroidea and are found to be closely related to both *Epimeria frankei* Beermann & Raupach, 2018 in Beerman, Westbury, Hofreiter, Hilgers, Deister, Neumann & Raupach, 2018 and *Epimeria cornigera* Fabricius, 1779 (family Epimeriidae Boeck, 1871) from the Iphimedioidea Boeck, 1871 superfamily. Similarly, *Charcotia amundseni* from family Lysianassidae Dana, 1849 is clustering together with *Eurythenes magellanicus* H. Milne Edwards, 1848 and *Eurythenes maldoror d'Udekem d'Acoz & Havermans, 2015* (family Eurytheneidae Stoddart & Lowry, 2004), *Hirondellea gigas* Birstein & Vinogradov, 1955 (family Hirondelleidae Lowry & Stoddart, 2010), and *Onisimus nanseni* G.O. Sars, 1900 (family Uristidae Hurley, 1963) and all belonging to the superfamily Lysianassoidea.

## Discussion

In the current study, we have assembled and annotated novel complete mitogenomes from two Antarctic amphipod species with a low coverage skimming sequencing approach. We have obtained very low percentages of ambiguities (<0.01%) illustrating that this cost efficient approach is very successful. Besides our study, only two complete mitogenomes from amphipods of the polar regions are currently available, namely, from *Gondogeneia antarctica* Chevreux 1905 from Antarctica (Shin et al. 2012) and *Onisimus nanseni* G.O. Sars, 1900 from the Arctic (Ki et al. 2010). Our study thus provides important novel genomic data for further research and the first complete mitogenomes of the widely spread amphipod genera *Eusirus* and *Charcotia*. Our comparisons of mitogenomes between two genetic clades, possibly resembling two different genetic species of *E.* cf. *giganteus* illustrate that mitogenomic features such as length, gene order, AT content, and tRNA structure are similar at the intraspecific level (Table 2, Supplementary Figure 1, Supplementary Figure 2; Supplementary Table 3, Supplementary Figure 4a and b).

All three newly obtained mitogenomes are with 15,534 and 15,619 bp at the middle range of reported lengths of published amphipod mitogenomes (14,113bp to 18,424 bp) (Romanova et al. 2016; Li et al. 2019b). The observed AT-richness of the mitogenomes of the current study (61.9% and 68.7%) is slightly lower than in other studies based on complete (Table 2) and incomplete amphipod mitogenomes where AT range between 69.79% and 74.35% (Li et al. 2019a). However our data are in line with Wilson et al. (2000) reporting such an AT-rich bias as typical for arthropods.

The negative GC skews on both strands of the protein coding genes of the two species in this study differ from the so far known common Malacostraca pattern where genes encoded on the + strand usually exhibit negative and genes encoded on the − strand positive GC skews (Hassanin 2006). The strand bias in nucleotide composition of metazoan mitogenomes is attributed to varying mutational pressure during replication or transcription (Pons et al. 2014; Hassanin et al. 2005). Future research will need to test if these factors are responsible for the different GC patterns observed in the two Antarctic amphipod species of the current study.

### Gene order and rearrangements

The translocations of *trnG* and a commonly derived pattern of a gene string consisting of *trnA*, *trnS1*, *trnN*, *trnE*, and *trnR* are presumed to be apomorphic features of certain amphipods (Kilpert and Podsiadlowski 2010; Krebes and Bastrop 2012; Li et al. 2019a). The two studied species exhibit the translocation of *trnG* relative to the pancrustacean ground pattern. However, the altered tRNA gene order of the two species results in a unique tRNA string that is dissimilar to the apomorphic gene string of *trnA*, *trnS1*, *trnN*, *trnE*, and *trnR*. Moreover, the observed *rrnL*-*trnV*-*rrnS* pattern of *E.* cf. *giganteus* is known to be common in most Malacostraca (Ki et al. 2010) and is also observed in the pancrustacean ground pattern. This is, however, not the case for *C. amundseni* with the *trnC* being present. In addition, the large-scale gene reversals that have been found in three species have also been observed in *Halice* sp. Boeck, 1871 (Li et al. 2019a). It may be attributed to intramitochondrial recombination allowing breaking and rejoining of the mitochondrial genome (Dowton and Austin 1999; Li et al. 2019a).

### rRNA genes

The shortest complete *rrnL* in amphipods of 577 bp is currently known from *Hirondellea gigas* Birstein & Vinogradov, 1955 (Lan et al. 2016), which is much shorter than the *rrnL* that has been found in the species of the current study. On the other hand, the *rrnS* of *C. amundseni* has with 529 bp the same length as *Alicella gigantea* (Li et al. 2019b) which has so far been the shortest reported *rrnS* length in amphipods. Also, the shortest total length of *rrnL* and *rrnS* together has been described from the amphipod *Hirondellea gigas* Birstein & Vinogradov, 1955 (Lan et al. 2016) with 1120 bp, and we find that the total length of the two rRNAs in *C. amundseni* is with 1268 bp rather similar. Short rRNA genes have also been observed in *Gammarus duebeni* Lilljeborg, 1852 (Krebes and Bastrop 2012) where they have been attributed to a minimization strategy of the mitogenome.

### tRNA secondary structures

Aberrant tRNA structures as we find them in the three novel mitogenomes are common. Jühling et al. (2012) have described a loss of a D-domain in *trnS1* in almost all metazoan, while the D-domain in *trnS2* has only been lacking in Lophotrochozoa and Ecdysozoa. Mitogenome studies of other amphipod species also report the lack of the DHU arm in *trnS1* and *trnS2* in *Epimeria cornigera* Fabricius, 1779, *Epimeria frankei* Beermann & Raupach, 2018 in Beerman, Westbury, Hofreiter, Hilgers, Deister, Neumann & Raupach, 2018 (Beermann et al. 2018), *Caprella scaura* Templeton, 1836 (Ito et al. 2010) and ‘Metacrangonyx boveii’ (Pons et al. 2014) and in *trnV* in *Brachyuropus grewingkii* Dybowsky, 1874, *Acanthogammarus victorii* Dybowsky, 1874, *Eulimnogammarus cyaneus* Dybowsky, 1874, and *Garjajewia cabanisii* Dybowsky, 1874 (Romanova et al. 2016), *Halice* sp. Boeck, 1871 (Li et al. 2019a) and ‘Metacrangonyx boveii’ (Pons et al. 2014). The absence of the TΨC loop is another aberrant and common structure in amphipod that has also been observed in *trnC*, *trnE*, and *trnT* of *Caprella mutica* Schurin, 1935 (Kilpert and Podsiadlowski 2010), *trnQ* and *trnV* of *Gammarus duebeni* Lilljeborg, 1852 (Krebes and Bastrop 2012), and *trnC*, *trnQ*, *trnK*, and *trnF* of *Onisimus nanseni* G.O. Sars, 1900 (Ki et al. 2010). The pressure for minimization of the mitogenome has been put forward as one of the explanations for these aberrant tRNA structures (Yamazaki et al. 1997). Other explanations could be replication slippage resulting in sequence deletions or insertions (Macey et al. 1997). Despite these aberrant structures in tRNAs, these are most likely still functional (Watanabe et al. 2014).

### Nucleotide diversity

Information on nucleotide diversity can be helpful for the design of new molecular markers (Romanova et al. 2016; Zhang et al. 2018). Here, we have shown that the most variable mitogenes of *Eusirus* for intraspecific comparisons between genetic clades are *nad6*, *nad5*, and *nad1* while for comparisons between *Eusirus* and *Charcotia, atp8*, *nad6,* and *nad2* are most variable, which could be suitable for future phylogeographic and population genetic studies. Contrary, the least variable mitogenes for *Eusirus* are *nad4*, *nad3*, *nad2*, and *cox1* and for interspecies comparisons between *Eusirus* and *Charcotia cox1*, *cytb*, and *cox3* could be more suitable for future deep phylogeny investigations. Surprisingly, despite its wide use in DNA barcoding initiatives (Witt et al. 2006; Hebert et al. 2003), the *cox1* gene appears to have relatively low nucleotide diversities between closely and distantly related amphipods. Consistent with our results, also Romanova et al. (2016) describe the mitogenes *atp8*, *nad2*, *nad4l*, *nad5*, and *nad6* as most variable in Baikalian amphipods and *cox* genes to be less variable, with *cox1* having the lowest nucleotide diversity.

### Phylogenetic analysis

Our evolutionary tree (Figure 1) constructed from mitochondrial protein coding genes is well supported and shows phylogenetic clades according to amphipod superfamily identity. Moreover, our results are congruent to the current taxonomic classification where the species were categorized into their respective family and superfamily (Horton et al. 2021). Previous classification have placed *Eusirus* in the same Eusiroidea superfamily as *Epimeria* (Bousfield 1978) while the recent classification have placed *Eusirus* under superfamily Eusiroidea and *Epimeria* under Iphimedioidea (Lowry and Myers 2017). Phylogenetic evidence using 18S rDNA have shown that *Eusirus* has a close relationship with Epimeria which showed a well-supported clade of Eusiridae, Calliopiidae, Astyridae, Iphimediidae, Epimeriidae, and Pleustidae families (Englisch 2001). Phylogenetic evidence using 13-protein coding genes further corroborates these close relationships (Figure 1).

Our grouping of *Charcotia amundseni* with other species from the superfamily Lysianassoidea (Figure 1) is supported with the morphological phylogeny of Lowry and Myers (2017), which characterized this superfamily as often having a type 3 lysianassoid calceolus and a cleft telson. Molecular phylogenetic analyses using concatenated 16S-COX1-18S data in Ritchie et al. (2015) show clustering of families and superfamilies similar to our study which further backs up our results. The phylogenetic grouping of the two morphospecies invested here based on the three novel mitogenomes thus follow the expected patterns according to taxonomic relationships.

## Conclusions

The current study provides three additional novel complete mitogenomes of Antarctic amphipod species and the first complete mitogenomes of the abundant amphipod genera *Eusirus* and *Charcotia*. In comparison to other published amphipod mitogenomes, the novel mitogenomes show distinct features such as a lower AT-richness in their whole mitogenomes, negative GC skews on both strands of the protein coding genes, and unique gene rearrangements. The novel mitogenomes also share characteristics with other amphipod mitogenomes including aberrant tRNA and short rRNA genes, which could be linked to minimalization of mitogenomes. Moreover, the estimation of the nucleotide diversity (π) provides information to choose mitogenes as most suitable markers for future phylogenetic studies of amphipods. The novel mitogenomes are certainly useful for future phylogenetic analyses as put the investigated species into phylogenetic positions matching superfamily and family identity.

## Supplementary Material

Supplemental Material

## Data Availability

The genome sequence data that support the findings of this study are openly available in GenBank of NCBI at https://www.ncbi.nlm.nih.gov/ under the accession numbers *Eusirus* cf. *giganteus* (G1) (OK489458)*, Eusirus* cf. *giganteus* (G2) (OK489459), and *Charcotia amundseni* (OK489457). The associated Bioproject is PRJNA769065 and the Biosample numbers are *Eusirus* cf. *giganteus* (G1) (SAMN22086850), *Eusirus* cf. *giganteus* (G2) (SAMN22087742), and *Charcotia amundseni* (SAMN22087745). The SRA accession numbers are *Eusirus* cf. *giganteus* (G1) (SRX13936485), *Eusirus* cf. *giganteus* (G2) (SRX13936486), and *Charcotia amundseni* (SRX13936484).
