# Supplementary material for: Describing novel mitochondrial genomes of Antarctic amphipods
Source: Mitochondrial DNA B Resour. 2022 May 10;7(5):810–8. doi: 10.1080/23802359.2022.2073837 (PMC9103263; doi:10.1080/23802359.2022.2073837)
Supplement: Supplemental Material [file TMDN_A_2073837_SM2152.docx]

Supplementary table 1. List of amphipod species species analysed for their AT and GC skew of mitogenomes. Target species of the current study are indicated in bold.

| Species | Family | Genbank No. | Publication |
| --- | --- | --- | --- |
| ***Eusirus* cf. *giganteus (G1)*** | **Eusiridae** | **OK489458** | **This study** |
| ***Eusirus* cf. *giganteus (G2)*** | **Eusiridae** | **OK489459** | **This study** |
| ***Charcotia amundseni*** | **Lysianassidae** | **OK489457** | **This study** |
| *Alicella gigantea* | Alicellidae | MK215211 | Li et al., 2019 |
| *Ampithoe lacertosa* | Ampithoidae | MK215645 | Lee et al, 2019 |
| *Bahadzia jaraguensis* | Hadziidae | FR872382 | Bauzà et al., 2012 |
| *Brachyuropus grewingkii* | Acanthogammaridae | KP161875 | Romanova et al., 2016 |
| *Caprella mutica* | Caprellidae | GU130250 | Kilpert et al., 2010 |
| *Caprella scaura* | Caprellidae | AB539699 | Ito et al., 2010 |
| *Epimeria cornigera* | Epimeriidae | MF361127 | Beerman, et al, 2018 |
| *Eulimnogammarus cyaneus* | Eulimnogammaridae | KX341964 | Romanova et al., 2016 |
| *Eulimnogammarus verrucosus* | Eulimnogammaridae | KF690638 | Rivarola-Duarte et al., 2014 |
| *Eulimnogammarus vittatus* | Eulimnogammaridae | KM287572 | Romanova et al., 2016 |
| *Eurythenes magellanicus* | Eurytheneidae | MN688221 | Li et al., 2020 |
| *Eurythenes maldoror* | Eurytheneidae | NC036429 | Cheng et al., unpublished |
| *Gammarus duebeni* | Gammaridae | NC_017760 | Krebes et al., 2012 |
| *Gammarus fossarum* | Gammaridae | NC_034937 | Macher et al., 2017 |
| *Gammarus lacustris* | Gammaridae | MK354235 | Sun et al, 2020 |
| *Gammarus pisinnus* | Gammaridae | MK354236 | Sun et al, 2020 |
| *Gammarus roeselii* | Gammaridae | MG779536 | Cormier et al, 2018 |
| *Gmelinoides fasciatus* | Micruropodidae | NC_033361 | Romanova et al., 2016 |
| *Gondogeneia antarctica* | Pontogeneiidae | JN827386 | Shin et al., 2012 |
| *Grandidierella fasciata* | Aoridae | LC500464 | Hiki et al, 2020 |
| *Grandidierella japonica* | Aoridae | LC500462 | Hiki et al, unpublished |
| *Grandidierella osakaensis* | Aoridae | LC546828 | Hiki et al., 2020 |
| *Grandidierella rubroantennata* | Aoridae | LC500463 | Hiki et al, 2020 |
| *Haploginglymus sp.* | Niphargidae | LT594768 | Pons et al., unpublished |
| *Hyalella azteca* | Hyalellidae | NC_039403 | Patra et al., unpublished |
| *Metacrangonyx boveei* | Metacrangonyctidae | HE860498 | Bauzà-Ribot et al., 2012 |
| *Metacrangonyx longipes* | Metacrangonyctidae | AM944817 | Bauzà-Ribot et al., 2009 |
| *Metacrangonyx nicoleae tamri* | Metacrangonyctidae | HE860504 | Bauzà-Ribot et al., 2012 |
| *Metacrangonyx repens* | Metacrangonyctidae | HE860495 | Bauzà-Ribot et al., 2012 |
| *Metacrangonyx spinicaudatus* | Metacrangonyctidae | HE860506 | Bauzà-Ribot et al., 2012 |
| *Onisimus nanseni* | Uristidae | FJ555185 | Ki et al., 2010 |
| *Pallaseopsis kesslerii* | Pallaseidae | KX341968 | Romanova et al., 2016 |
| *Platorchestia japonica* | Talitridae | MG010370 | Yang et al., 2017 |
| *Platorchestia parapacifica* | Talitridae | MG010371 | Yang et al., 2017 |
| *Pleonexes koreana* | Ampithoidae | MK265245 | Lee et al, 2019 |
| *Pseudocrangonyx daejeonensis* | Pseudocrangonyctidae | MH229998 | Lee et al., 2018 |
| *Pseudoniphargus daviui* | Pseudoniphargidae | FR872383 | Stokkan et al., 2016 |
| *Stygobromus indentatus* | Crangonyctidae | KU869711 | Aunins et al., 2016 |
| *Stygobromus tenuis potomacus* | Crangonyctidae | KU869712 | Aunins et al., 2016 |
| *Trinorchestia longiramus* | Talitridae | MH542431 | Patra et al., 2019 |

Supplementary table 2. Annotation of the complete mitogenomes of *Eusirus* cf. *giganteus* (G1 and G2) and *Charcotia amundseni*

| ***Eusirus* cf. *giganteus* (G1)** | | | | | | |
| --- | --- | --- | --- | --- | --- | --- |
| **Gene** | **Strand** | **From** | **To** | **Length (bp)** | **Start** | **Stop** |
| *cox1* | + | 1 | 1537 | 1537 | ATT | T(AA) |
| *trnL2* | + | 1538 | 1601 | 64 |  |  |
| *cox2* | + | 1602 | 2279 | 678 | ATC | TAA |
| Intergenic spacer 1 |  | 2280 | 2280 | 1 |  |  |
| *trnK* | + | 2281 | 2339 | 59 |  |  |
| Intergenic spacer 2 |  | 2340 | 2412 | 73 |  |  |
| *trnD* | + | 2413 | 2477 | 65 |  |  |
| *atp8* | + | 2478 | 2636 | 159 | ATC | TAA |
| *atp6* | + | 2630 | 3300 | 671 | ATG | TA(A) |
| *cox3* | + | 3300 | 4088 | 789 | ATG | TAA |
| Intergenic spacer 3 |  | 4089 | 4100 | 12 |  |  |
| *nad3* | + | 4101 | 4448 | 348 | ATT | TAA |
| *trnA* | + | 4447 | 4504 | 58 |  |  |
| *trnS1* | + | 4504 | 4555 | 52 |  |  |
| Intergenic spacer 4 |  | 4556 | 4559 | 4 |  |  |
| *trnR* | + | 4560 | 4619 | 60 |  |  |
| Intergenic spacer 5 |  | 4620 | 4627 | 8 |  |  |
| *trnN* | + | 4628 | 4688 | 61 |  |  |
| *trnE* | + | 4686 | 4747 | 62 |  |  |
| Intergenic spacer 6 |  | 4748 | 4798 | 51 |  |  |
| *nad5* | - | 4799 | 6511 | 1713 | ATG | TAA |
| *trnH* | - | 6512 | 6572 | 61 |  |  |
| *nad4* | - | 6573 | 7878 | 1306 | ATG | T(AA) |
| Intergenic spacer 8 |  | 7879 | 7883 | 5 |  |  |
| *nad4l* | - | 7884 | 8177 | 294 | ATG | TAA |
| Intergenic spacer 9 |  | 8178 | 8182 | 5 |  |  |
| *trnT* | + | 8183 | 8242 | 60 |  |  |
| *trnP* | - | 8242 | 8303 | 62 |  |  |
| Intergenic spacer 10 |  | 8304 | 8305 | 2 |  |  |
| *nad6* | + | 8306 | 8881 | 576 | ATG | TAA |
| *nad1* | - | 8843 | 9763 | 921 | ATT | TAA |
| Intergenic spacer 11 |  | 9764 | 9860 | 97 |  |  |
| *trnM* | - | 9861 | 9920 | 60 |  |  |
| Intergenic spacer 12 |  | 9921 | 9933 | 13 |  |  |
| *cytb* | + | 9934 | 11067 | 1134 | ATG | TAG |
| *trnS2* | + | 11067 | 11118 | 52 |  |  |
| Intergenic spacer 13 |  | 11119 | 11204 | 86 |  |  |
| Putative control region |  | 11205 | 11458 | 254 |  |  |
| Intergenic spacer 14 |  | 11459 | 12306 | 848 |  |  |
| *rrnL* | - | 12307 | 13177 | 871 |  |  |
| Intergenic spacer 15 |  | 13178 | 13201 | 24 |  |  |
| *trnV* | - | 13202 | 13254 | 53 |  |  |
| *rrnS* | - | 13255 | 13935 | 681 |  |  |
| Intergenic spacer 16 |  | 13936 | 13976 | 41 |  |  |
| *trnI* | - | 13977 | 14042 | 66 |  |  |
| Intergenic spacer 17 |  | 14043 | 14051 | 9 |  |  |
| *trnY* | - | 14052 | 14110 | 59 |  |  |
| *trnQ* | - | 14107 | 14165 | 59 |  |  |
| Intergenic spacer 18 |  | 14166 | 14189 | 24 |  |  |
| *trnL1* | - | 14190 | 14253 | 64 |  |  |
| Intergenic spacer 19 |  | 14254 | 14280 | 27 |  |  |
| *trnC* | + | 14281 | 14339 | 59 |  |  |
| *trnF* | + | 14338 | 14397 | 60 |  |  |
| Intergenic spacer 20 |  | 14398 | 14445 | 48 |  |  |
| *nad2* | + | 14446 | 15433 | 988 | ATC | T(AA) |
| *trnW* | + | 15434 | 15497 | 64 |  |  |
| *trnG* | + | 15497 | 15558 | 62 |  |  |
|  |  |  |  |  |  |  |
| ***Eusirus* cf. *giganteus* (G2)** | | | | | | |
| **Gene** | **Strand** | **From** | **To** | **Length (bp)** | **Start** | **Stop** |
| *cox1* | + | 1 | 1537 | 1537 | ATT | T(AA) |
| *trnL2* | + | 1538 | 1601 | 64 |  |  |
| *cox2* | + | 1602 | 2279 | 678 | ATC | TAA |
| Intergenic spacer 1 |  | 2280 | 2280 | 1 |  |  |
| *trnK* | + | 2281 | 2339 | 59 |  |  |
| Intergenic spacer 2 |  | 2340 | 2412 | 73 |  |  |
| *trnD* | + | 2413 | 2477 | 65 |  |  |
| *atp8* | + | 2478 | 2636 | 159 | ATC | TAA |
| *atp6* | + | 2630 | 3300 | 671 | ATG | TA(A) |
| *cox3* | + | 3300 | 4088 | 789 | ATG | TAA |
| Intergenic spacer 3 |  | 4089 | 4100 | 12 |  |  |
| *nad3* | + | 4101 | 4448 | 348 | ATT | TAA |
| *trnA* | + | 4447 | 4504 | 58 |  |  |
| *trnS1* | + | 4504 | 4555 | 52 |  |  |
| Intergenic spacer 4 |  | 4556 | 4559 | 4 |  |  |
| *trnR* | + | 4560 | 4619 | 60 |  |  |
| Intergenic spacer 5 |  | 4620 | 4626 | 7 |  |  |
| *trnN* | + | 4627 | 4687 | 61 |  |  |
| *trnE* | + | 4685 | 4746 | 62 |  |  |
| Intergenic spacer 6 |  | 4747 | 4797 | 51 |  |  |
| *nad5* | - | 4798 | 6507 | 1710 | ATG | TAA |
| Intergenic spacer 7 |  | 6508 | 6509 | 2 |  |  |
| *trnH* | - | 6510 | 6572 | 63 |  |  |
| *nad4* | - | 6571 | 7853 | 1283 | ATG | TA(A) |
| Intergenic spacer 8 |  | 7854 | 7858 | 5 |  |  |
| *nad4l* | - | 7859 | 8152 | 294 | ATG | TAA |
| Intergenic spacer 9 |  | 8153 | 8157 | 5 |  |  |
| *trnT* | + | 8158 | 8217 | 60 |  |  |
| *trnP* | - | 8217 | 8278 | 62 |  |  |
| Intergenic spacer 10 |  | 8279 | 8280 | 2 |  |  |
| *nad6* | + | 8281 | 8818 | 538 | ATG | T(AA) |
| *nad1* | - | 8818 | 9738 | 921 | ATT | TAA |
| Intergenic spacer 11 |  | 9739 | 9835 | 97 |  |  |
| *trnM* | - | 9836 | 9895 | 60 |  |  |
| Intergenic spacer 12 |  | 9896 | 9908 | 13 |  |  |
| *cytb* | + | 9909 | 11042 | 1134 | ATG | TAG |
| *trnS2* | + | 11042 | 11093 | 52 |  |  |
| Intergenic spacer 13 |  | 11094 | 11179 | 86 |  |  |
| Putative control region |  | 11180 | 11433 | 254 |  |  |
| Intergenic spacer 14 |  | 11434 | 12282 | 849 |  |  |
| *rrnL* | - | 12283 | 13153 | 871 |  |  |
| Intergenic spacer 15 |  | 13154 | 13177 | 24 |  |  |
| *trnV* | - | 13178 | 13230 | 53 |  |  |
| *rrnS* | - | 13231 | 13911 | 681 |  |  |
| Intergenic spacer 16 |  | 13912 | 13952 | 41 |  |  |
| *trnI* | - | 13953 | 14018 | 66 |  |  |
| Intergenic spacer 17 |  | 14019 | 14027 | 9 |  |  |
| *trnY* | - | 14028 | 14086 | 59 |  |  |
| *trnQ* | - | 14083 | 14141 | 59 |  |  |
| Intergenic spacer 18 |  | 14142 | 14165 | 24 |  |  |
| *trnL1* | - | 14166 | 14229 | 64 |  |  |
| Intergenic spacer 19 |  | 14230 | 14256 | 27 |  |  |
| *trnC* | + | 14257 | 14315 | 59 |  |  |
| *trnF* | + | 14314 | 14373 | 60 |  |  |
| Intergenic spacer 20 |  | 14374 | 14421 | 48 |  |  |
| *nad2* | + | 14422 | 15409 | 988 | ATC | T(AA) |
| *trnW* | + | 15410 | 15473 | 64 |  |  |
| *trnG* | + | 15473 | 15534 | 62 |  |  |
|  |  |  |  |  |  |  |
| ***Charcotia amundseni*** | | | | | | |
| **Gene** | **Strand** | **From** | **To** | **Length (bp)** | **Start** | **Stop** |
| *cox1* | + | 1 | 1534 | 1534 | ATA | T(AA) |
| *trnL2* | + | 1535 | 1596 | 62 |  |  |
| *cox2* | + | 1597 | 2275 | 679 | ATG | T(AA) |
| *trnD* | + | 2276 | 2336 | 61 |  |  |
| Intergenic spacer 2 |  | 2337 | 2338 | 2 |  |  |
| *atp8* | + | 2339 | 2497 | 159 | ATA | TAA |
| *atp6* | + | 2491 | 3162 | 672 | ATG | TAA |
| *cox3* | + | 3162 | 3950 | 789 | ATG | TAA |
| Intergenic spacer 3 |  | 3951 | 3963 | 13 |  |  |
| *nad3* | + | 3964 | 4305 | 342 | ATT | TAA |
| Intergenic spacer 4 |  | 4306 | 4307 | 2 |  |  |
| *trnS1* | + | 4308 | 4362 | 55 |  |  |
| *trnN* | + | 4362 | 4428 | 67 |  |  |
| *trnE* | + | 4426 | 4485 | 60 |  |  |
| *trnF* | + | 4485 | 4538 | 54 |  |  |
| Intergenic spacer 5 |  | 4539 | 5273 | 735 |  |  |
| Putative control region |  | 5274 | 5839 | 566 |  |  |
| *nad5* | - | 5840 | 7558 | 1719 | ATG | TAA |
| *trnH* | - | 7559 | 7617 | 59 |  |  |
| *nad4* | - | 7618 | 8923 | 1306 | ATC | T(AA) |
| Intergenic spacer 7 |  | 8924 | 8928 | 5 |  |  |
| *nad4l* | - | 8929 | 9219 | 291 | ATA | TAG |
| Intergenic spacer 8 |  | 9220 | 9222 | 3 |  |  |
| *trnP* | - | 9223 | 9283 | 61 |  |  |
| Intergenic spacer 9 |  | 9284 | 9596 | 313 |  |  |
| *nad1* | - | 9597 | 10514 | 918 | ATG | TAA |
| Intergenic spacer 10 |  | 10515 | 10516 | 2 |  |  |
| *trnL1* | - | 10517 | 10575 | 59 |  |  |
| Intergenic spacer 11 |  | 10576 | 10587 | 12 |  |  |
| *rrnL* | - | 10588 | 11326 | 739 |  |  |
| Intergenic spacer 12 |  | 11327 | 11589 | 263 |  |  |
| *trnC* | - | 11590 | 11648 | 59 |  |  |
| *trnV* | - | 11647 | 11711 | 65 |  |  |
| *rrnS* | - | 11699 | 12227 | 529 |  |  |
| Intergenic spacer 13 |  | 12228 | 12335 | 108 |  |  |
| *trnQ* | - | 12336 | 12398 | 63 |  |  |
| *trnS2* | - | 12397 | 12449 | 53 |  |  |
| Intergenic spacer 14 |  | 12450 | 12456 | 7 |  |  |
| *cytb* | - | 12457 | 13596 | 1140 | ATG | TAA |
| *nad6* | - | 13596 | 14075 | 480 | ATG | TAA |
| Intergenic spacer 15 |  | 14076 | 14076 | 1 |  |  |
| *trnT* | - | 14077 | 14137 | 61 |  |  |
| *trnR* | - | 14132 | 14192 | 61 |  |  |
| Intergenic spacer 16 |  | 14193 | 14194 | 2 |  |  |
| *trnA* | - | 14195 | 14256 | 62 |  |  |
| Intergenic spacer 17 |  | 14257 | 14259 | 3 |  |  |
| *trnK* | - | 14260 | 14320 | 61 |  |  |
| Intergenic spacer 18 |  | 14321 | 14339 | 19 |  |  |
| *trnM* | + | 14340 | 14401 | 62 |  |  |
| *trnY* | - | 14400 | 14459 | 60 |  |  |
| *trnI* | + | 14458 | 14515 | 58 |  |  |
| *nad2* | + | 14516 | 15496 | 981 | ATA | TAA |
| *trnW* | + | 15495 | 15555 | 61 |  |  |
| *trnG* | + | 15556 | 15619 | 64 |  |  |

Supplementary table 3. AT content (%) in three codon positions of the mitochondrial protein coding genes in *E.* cf. *giganteus* (G1 and G2) and *C. amundseni*

| Species | First codon position | Second codon position | Third codon position |
| --- | --- | --- | --- |
| *Eusirus* cf. *giganteus* (G1) | 57.91 | 61.18 | 59.88 |
| *Eusirus* cf. *giganteus* (G2) | 57.89 | 61.12 | 59.87 |
| *Charcotia amundseni* | 64.08 | 65.19 | 72.61 |

Supplementary table 4. AT and GC skews of the protein-coding genes of *E.* cf. *giganteus* (G1), *E.* cf. *giganteus* (G2) and *C. amundseni*  encoded in the positive and negative strands

| Species | + | | - | |
| --- | --- | --- | --- | --- |
|  | **AT skew** | **GC skew** | **AT skew** | **GC skew** |
| *Eusirus* cf. *giganteus* (G1) | -0.144 | -0.143 | 0.220 | -0.315 |
| *Eusirus* cf. *giganteus* (G2) | -0.147 | -0.144 | 0.215 | -0.315 |
| *Charcotia amundseni* | -0.052 | -0.244 | 0.253 | -0.38 |


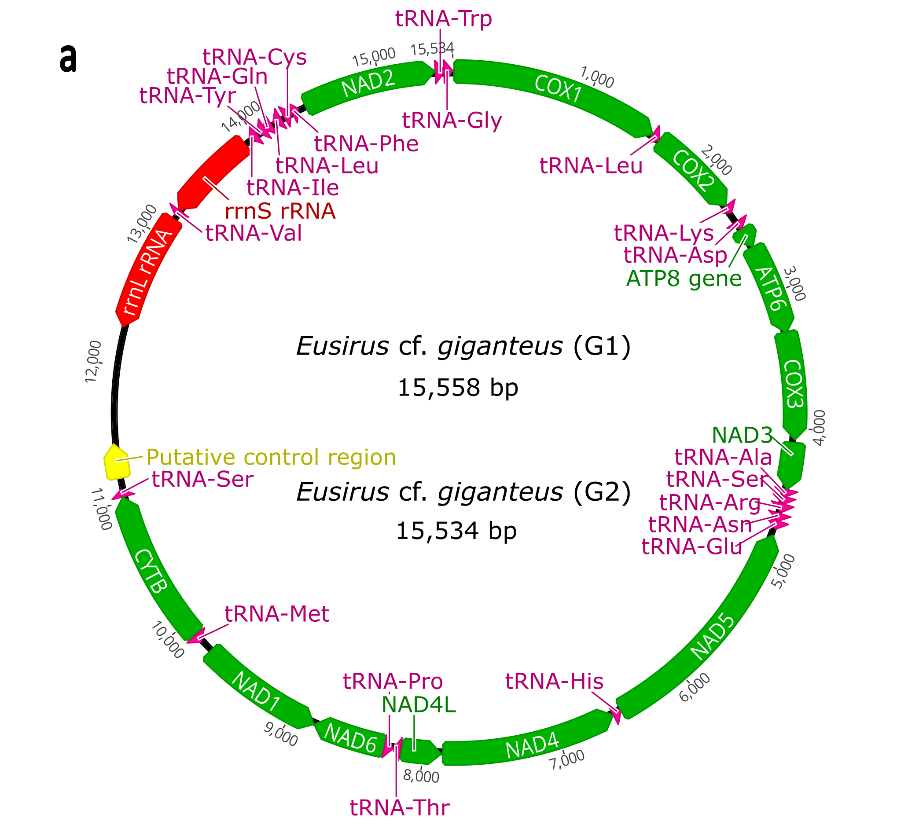

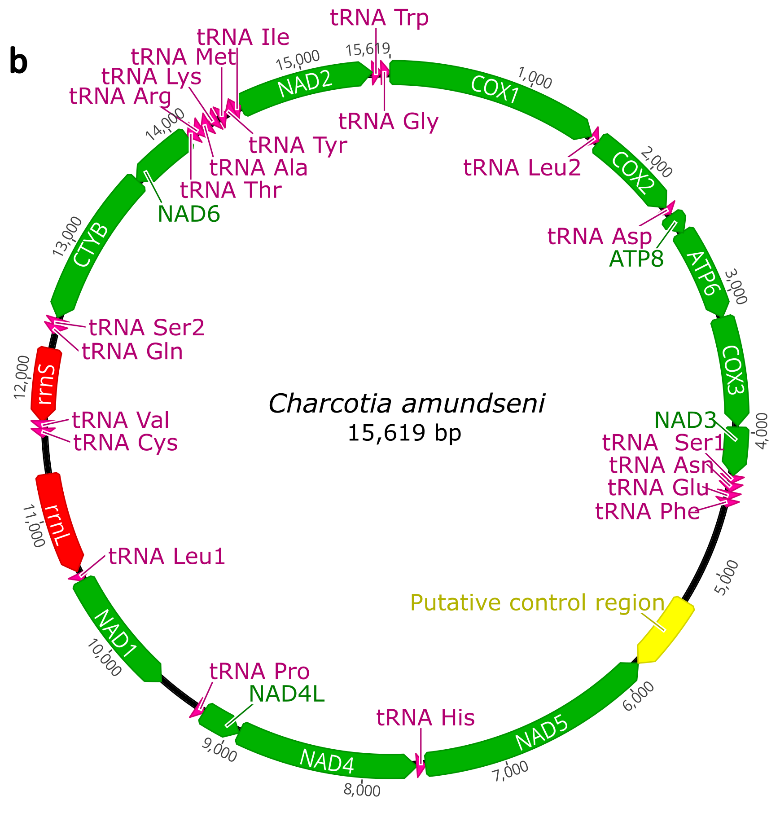


Supplementary figure 1. Structural organisation of the complete mitochondrial genome of **a** Eusirus cf. giganteus (G1) and Eusirus cf. giganteus (G2) **b** Charcotia amundseni. Protein coding genes are indicated in green, ribosomal RNAs (rRNAs) in red, transfer RNAs (tRNAs) in pink, and the putative control region (CR) in yellow. Direction of transcription is indicated by the arrowheads.


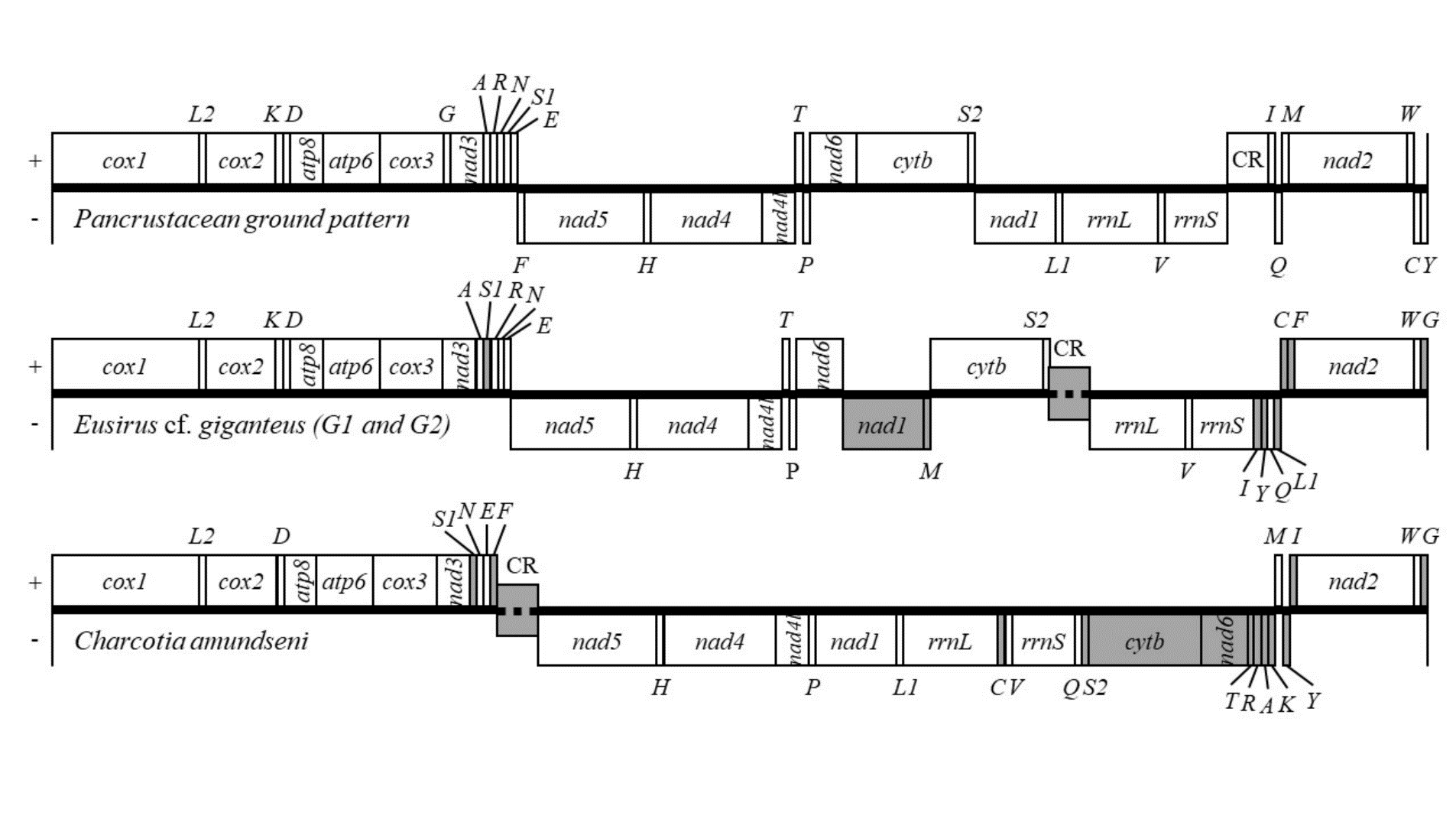


Supplementary figure 2. Organization of mitochondrial genomes of the three Antarctic amphipod species in this study in comparison to the putative pancrustacean ground pattern. Grey-colored genes indicate changes of gene order relative to the putative pancrustacean ground pattern. Genes on the (+) strand are found above the line while genes on the (-) strand are found below the line. tRNA genes are labeled using their single-letter amino acid code: G - Glycine, P - Proline, A - Alanine, V - Valine, L1 - Leucine1, L2 - Leucine2 , I - Isoleucine, M - Methionine, C - Cysteine, F - Phenylalanine, Y - Tyrosine, W - Tryptophan, H - Histidine, K - Lysine, R - Arginine, Q - Glutamine, N - Asparagine, E - Glutamic Acid, D - Aspartic Acid, S1 - Serine 1, S2 - Serine2, T - Threonine.

**a**

Family diagram for Pancrustacean ground pattern


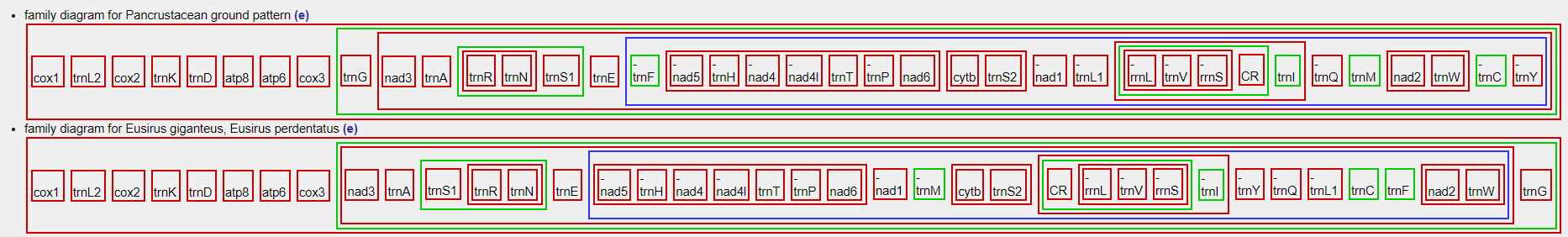


Family diagram for Eusirus giganteus (G1) and Eusirus giganteus (G2)


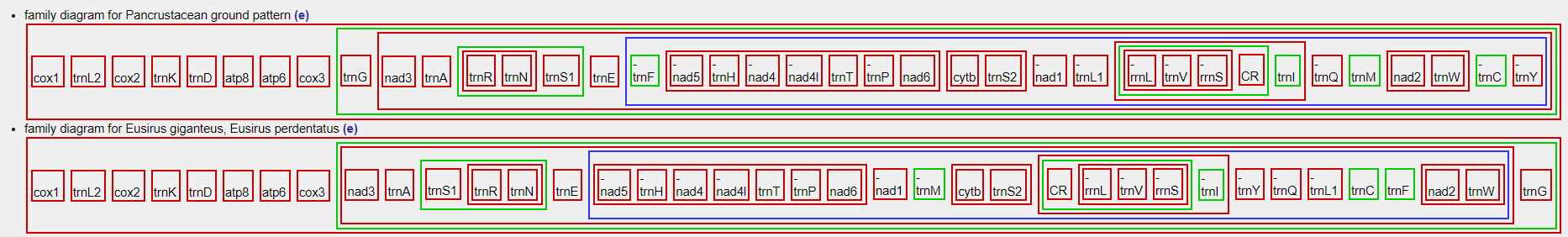


Scenario:

transposition


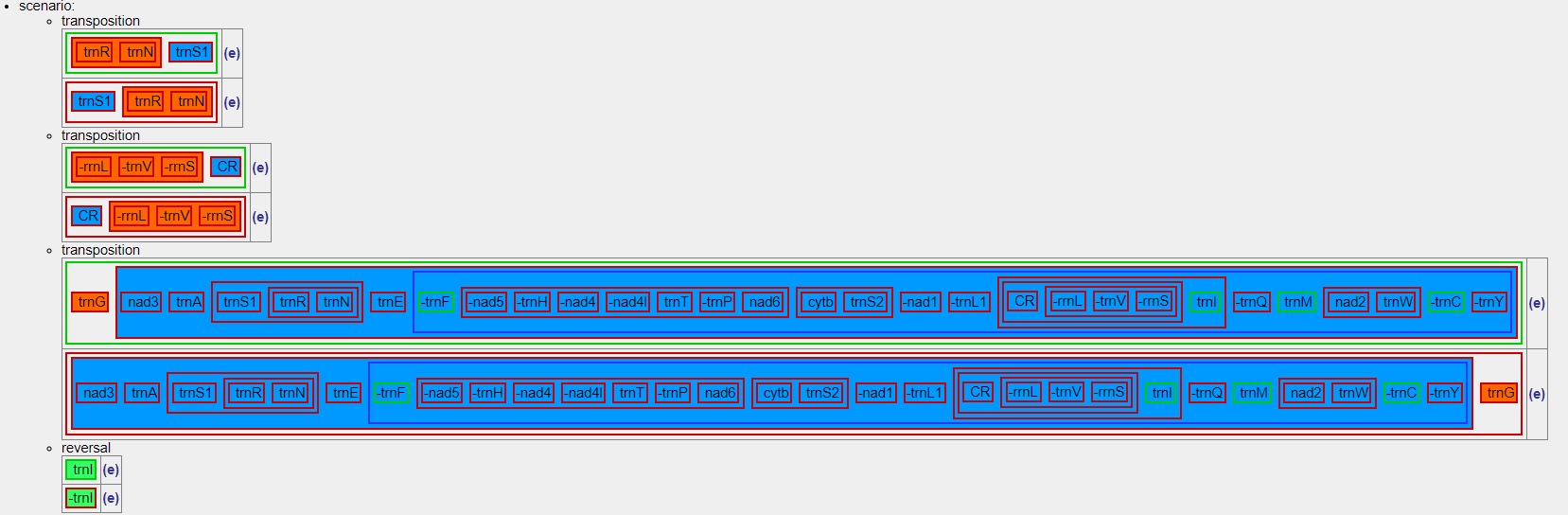


transposition


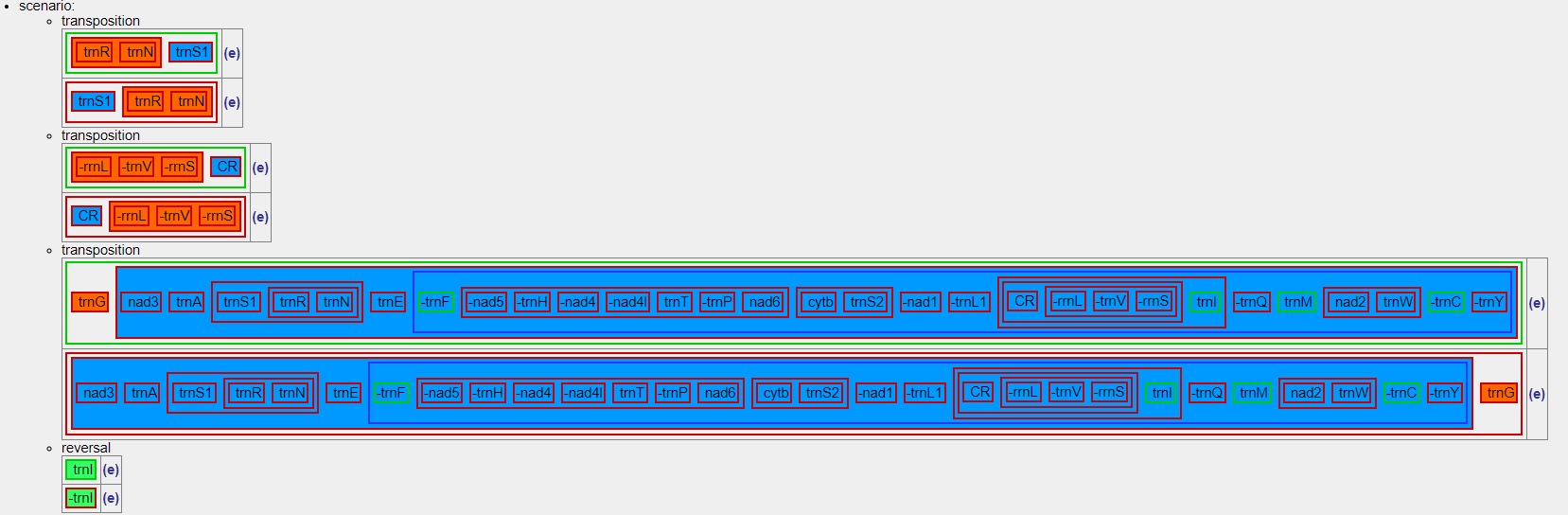


transposition
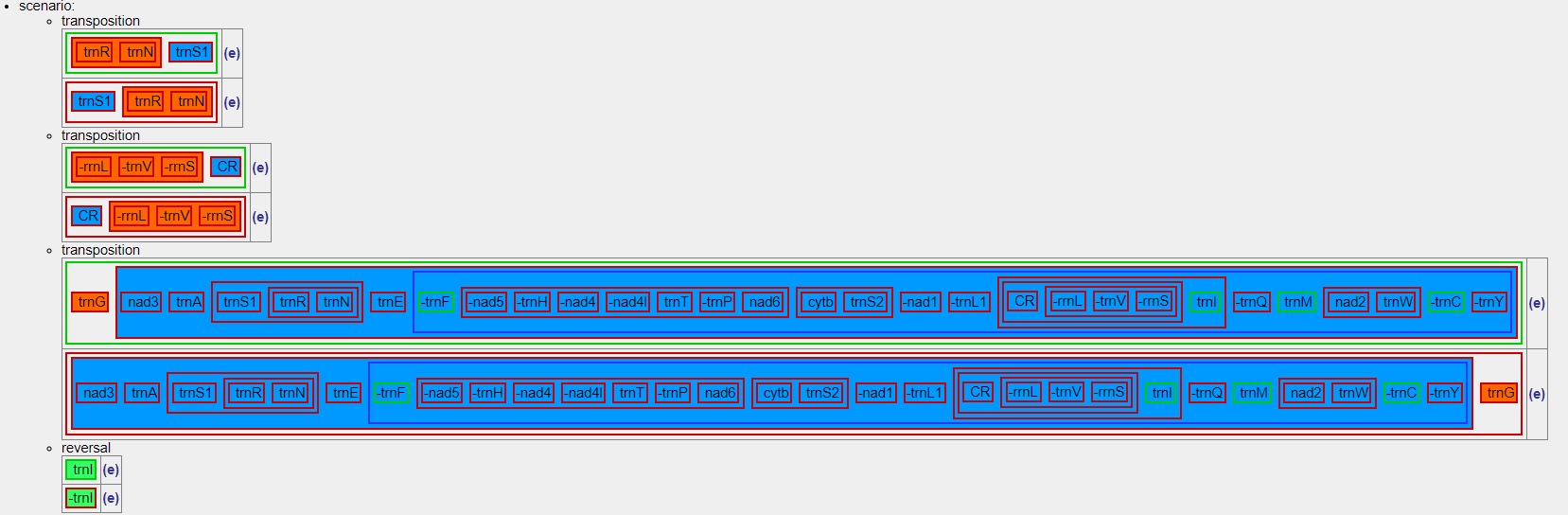


reversal


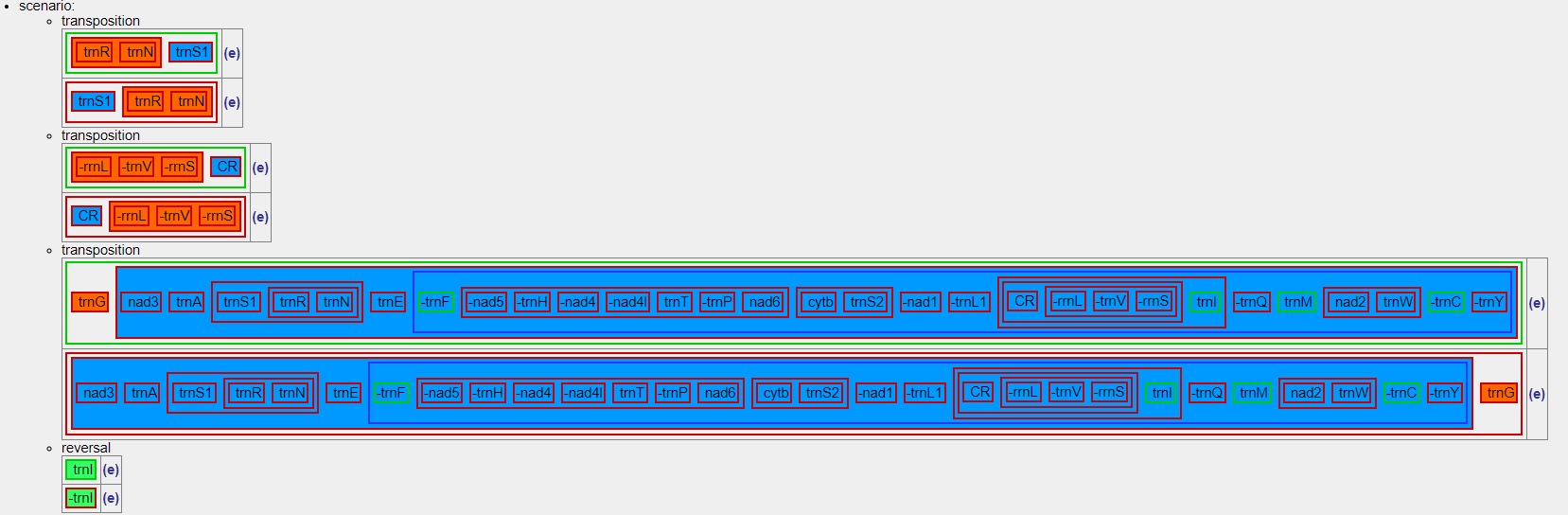


reversal


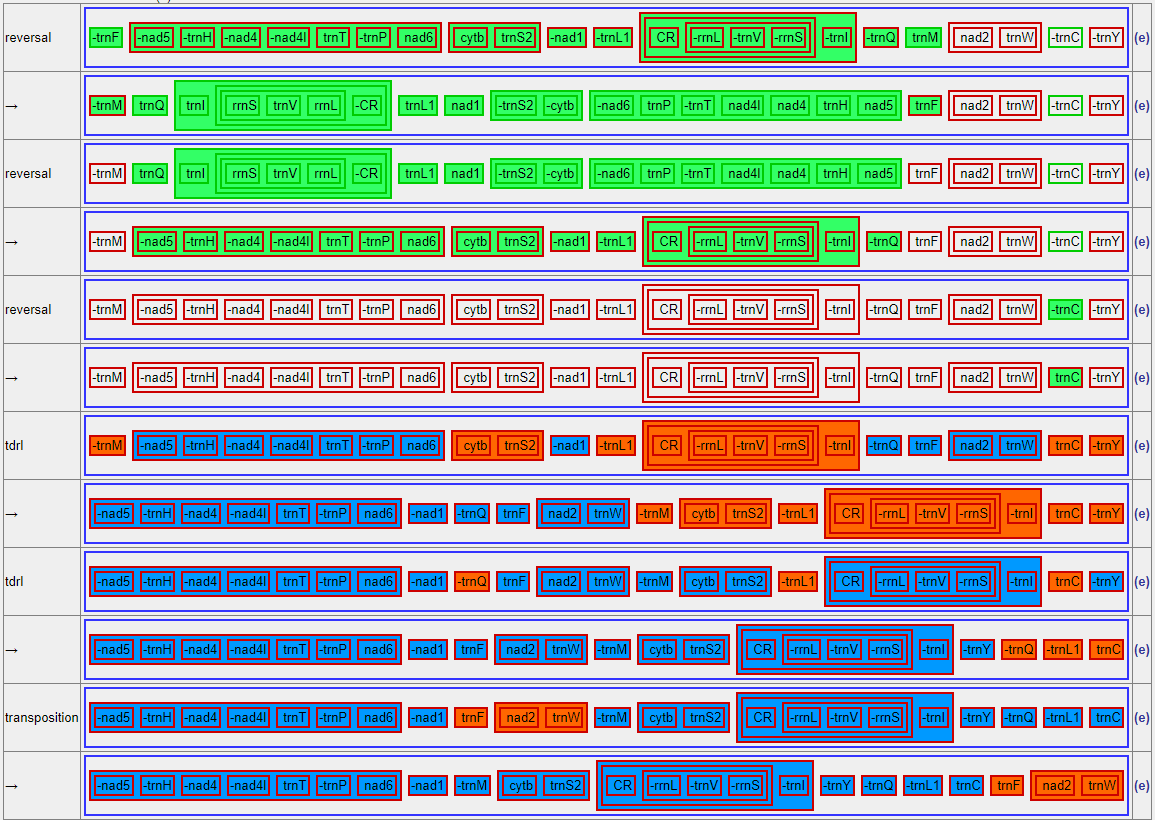


reversal


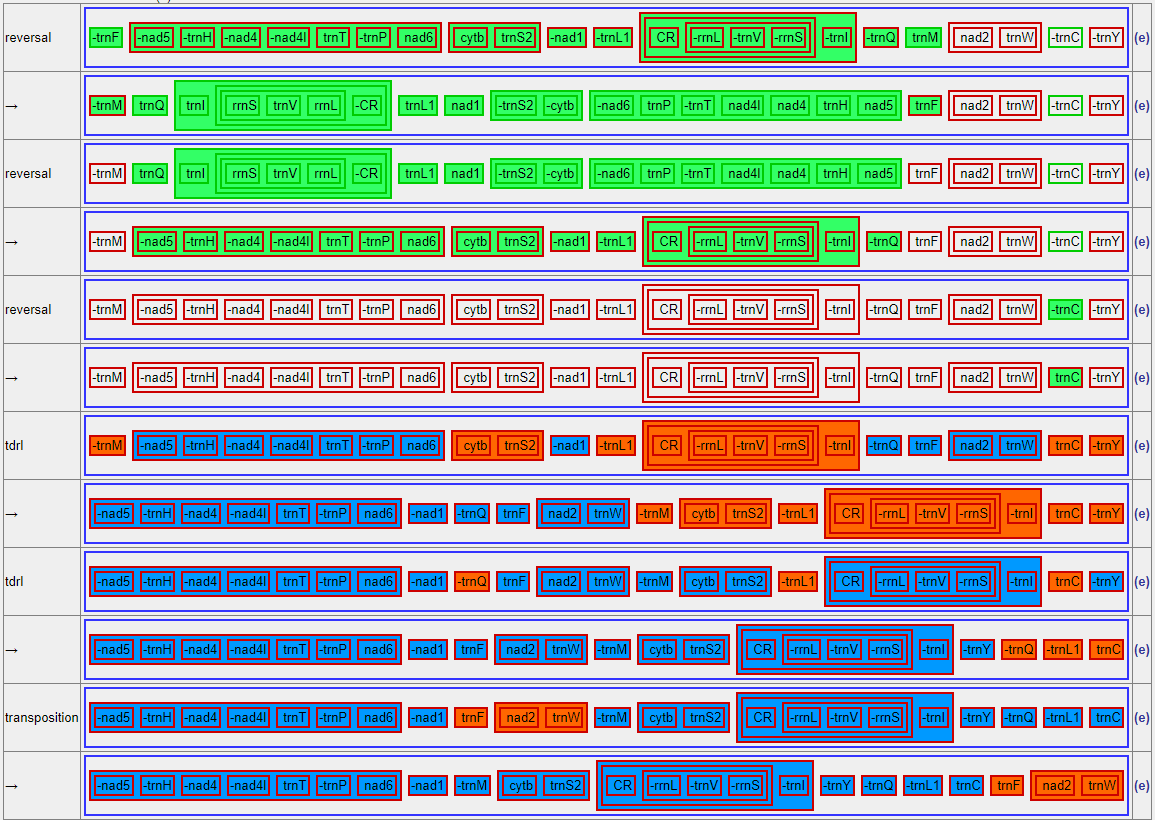


reversal


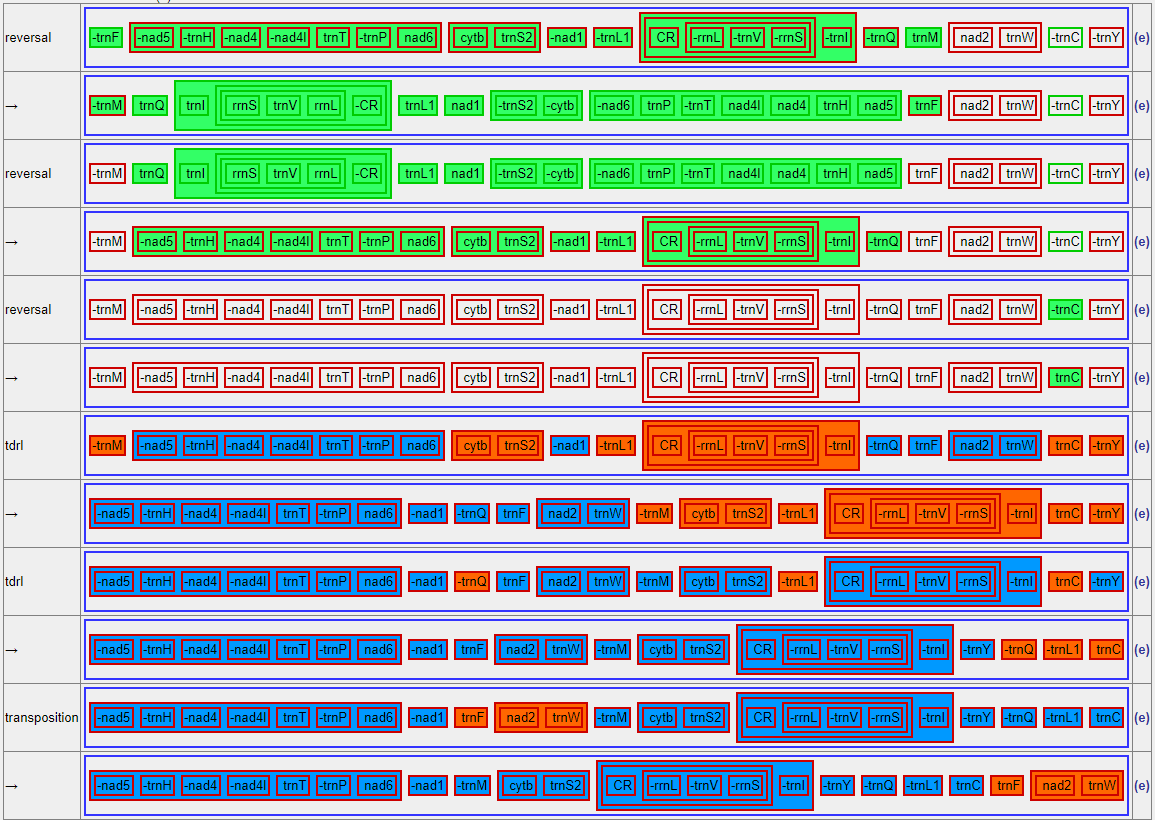


tdrl


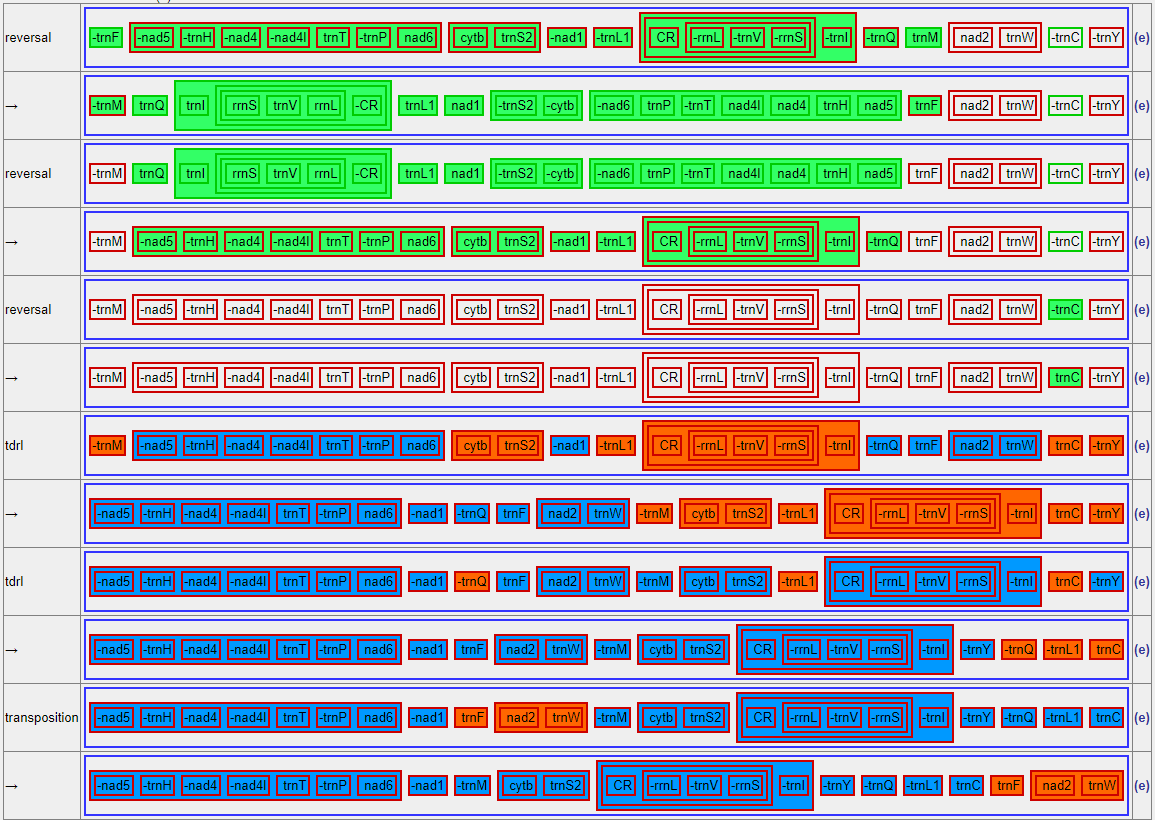


tdrl


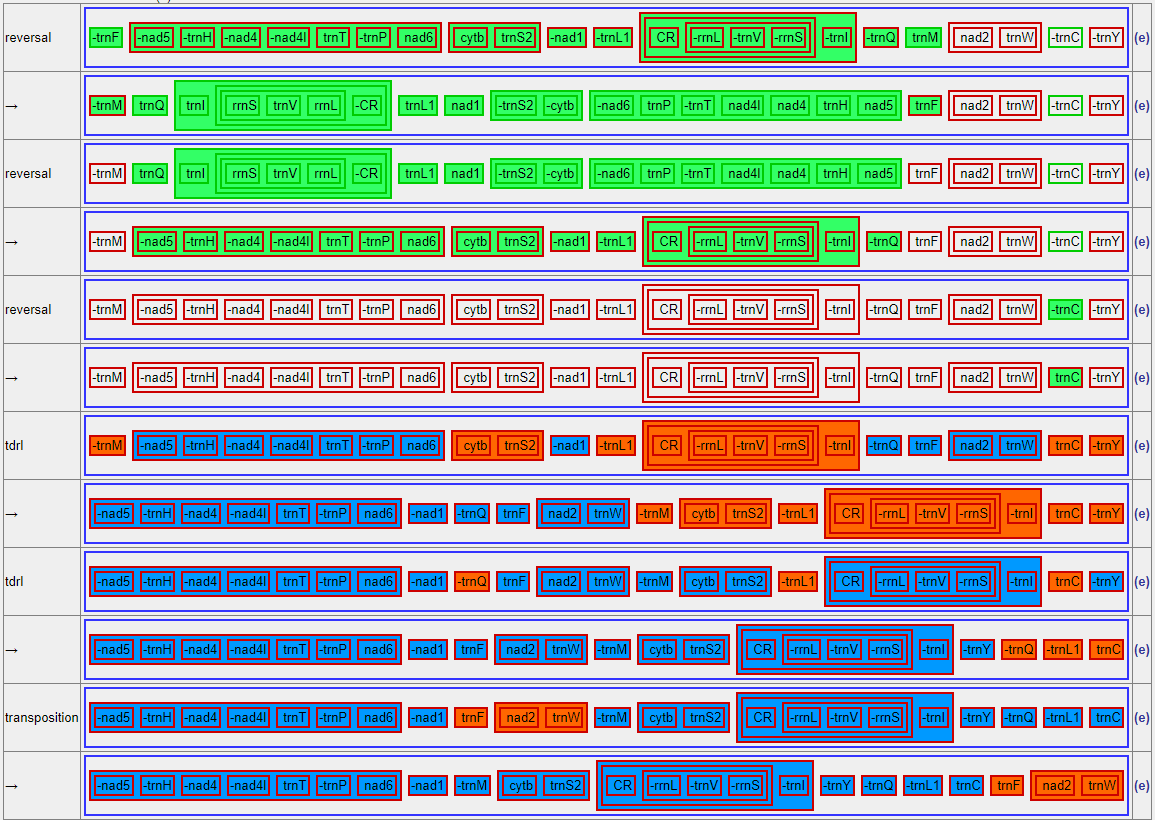


transposition


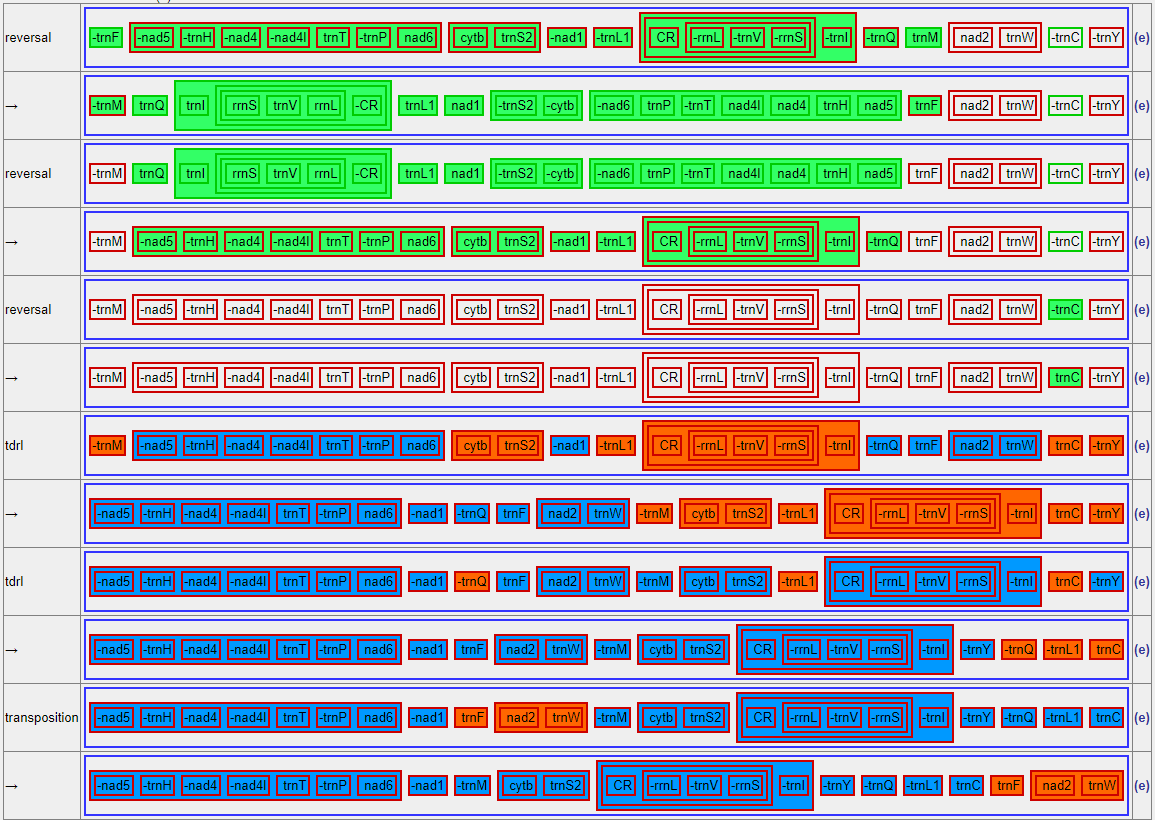


**b**

Family diagram for Pancrustacean ground pattern


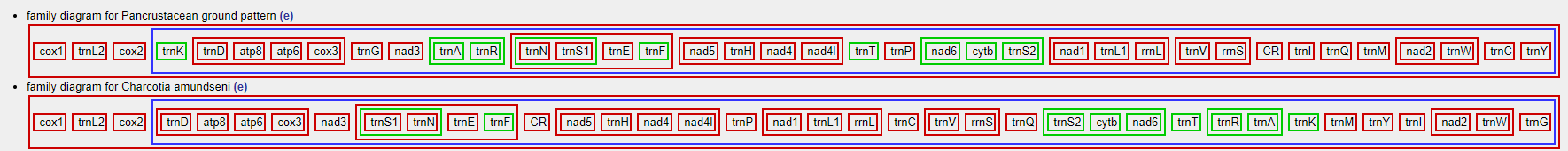


Family diagram for Charcotia amundseni


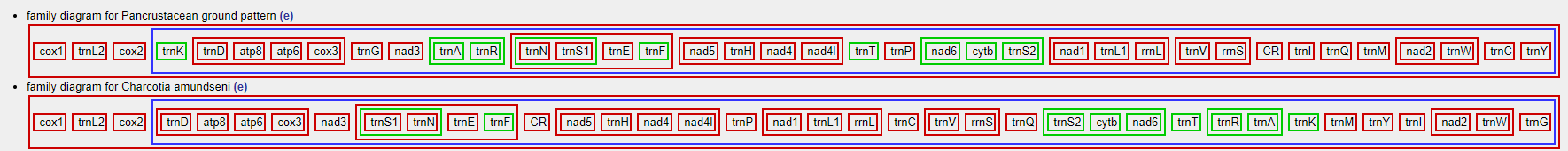


Scenario:

transposition


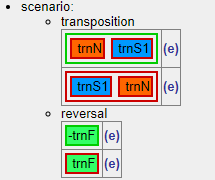


reversal


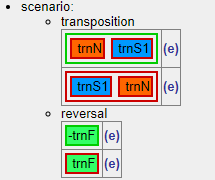


reversal


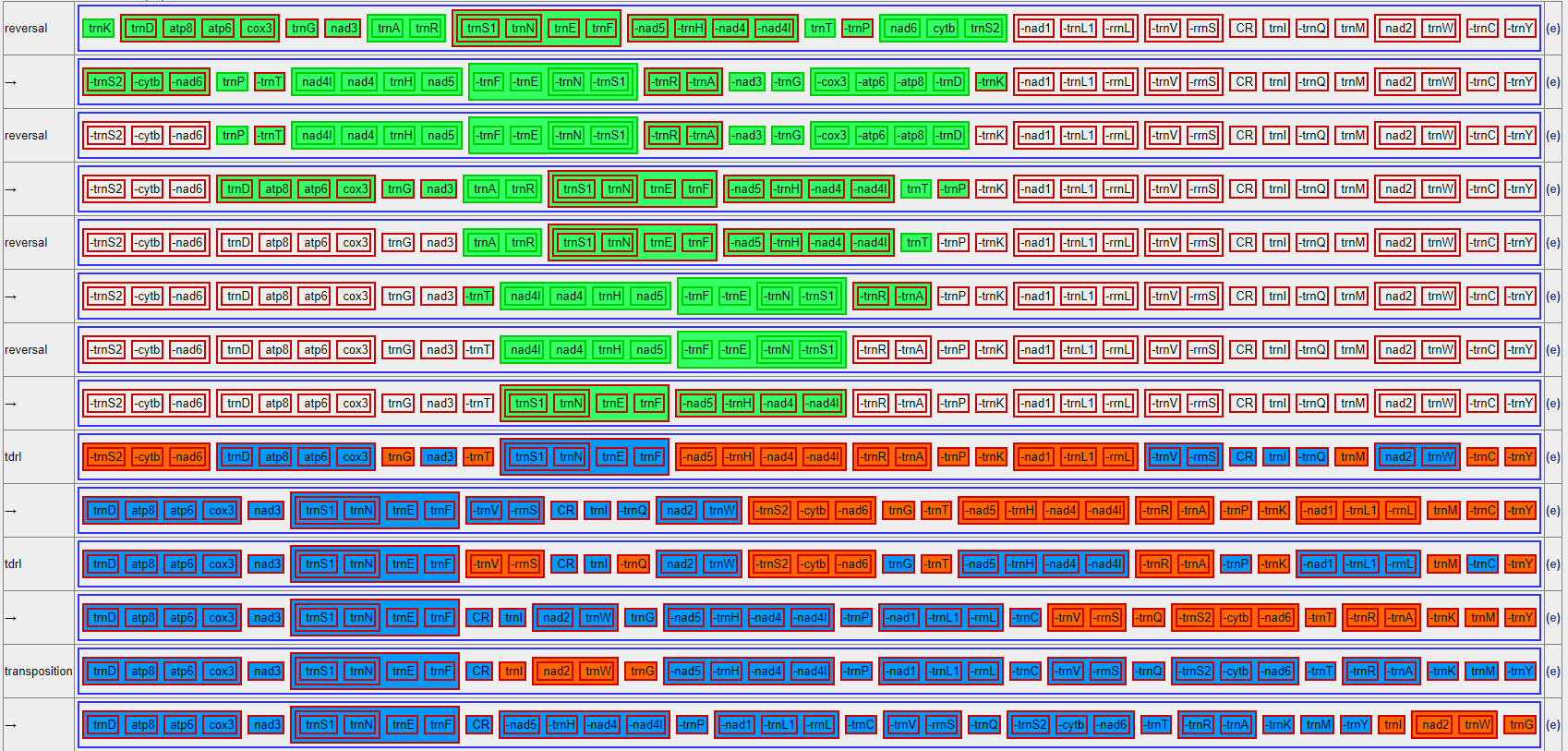


reversal


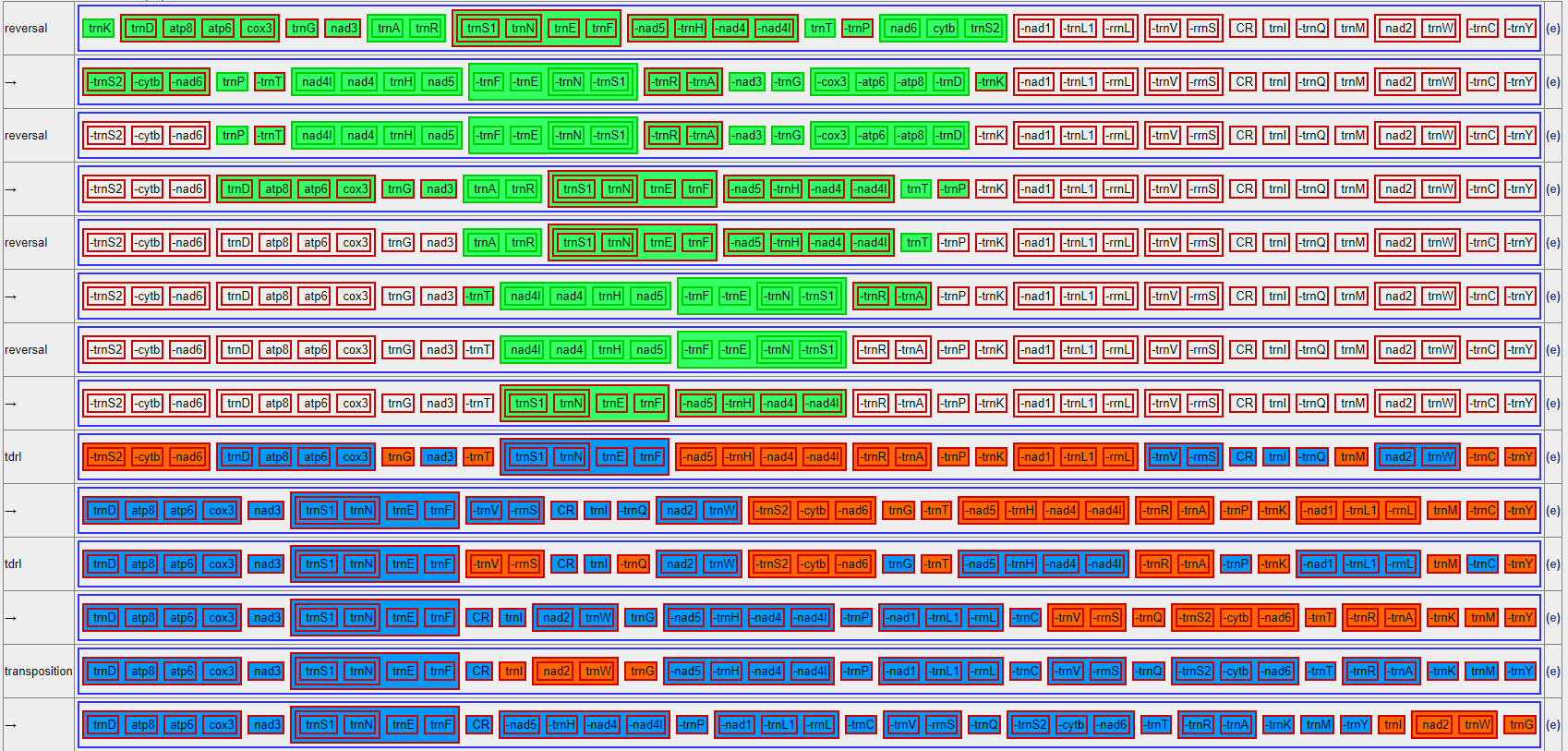


reversal


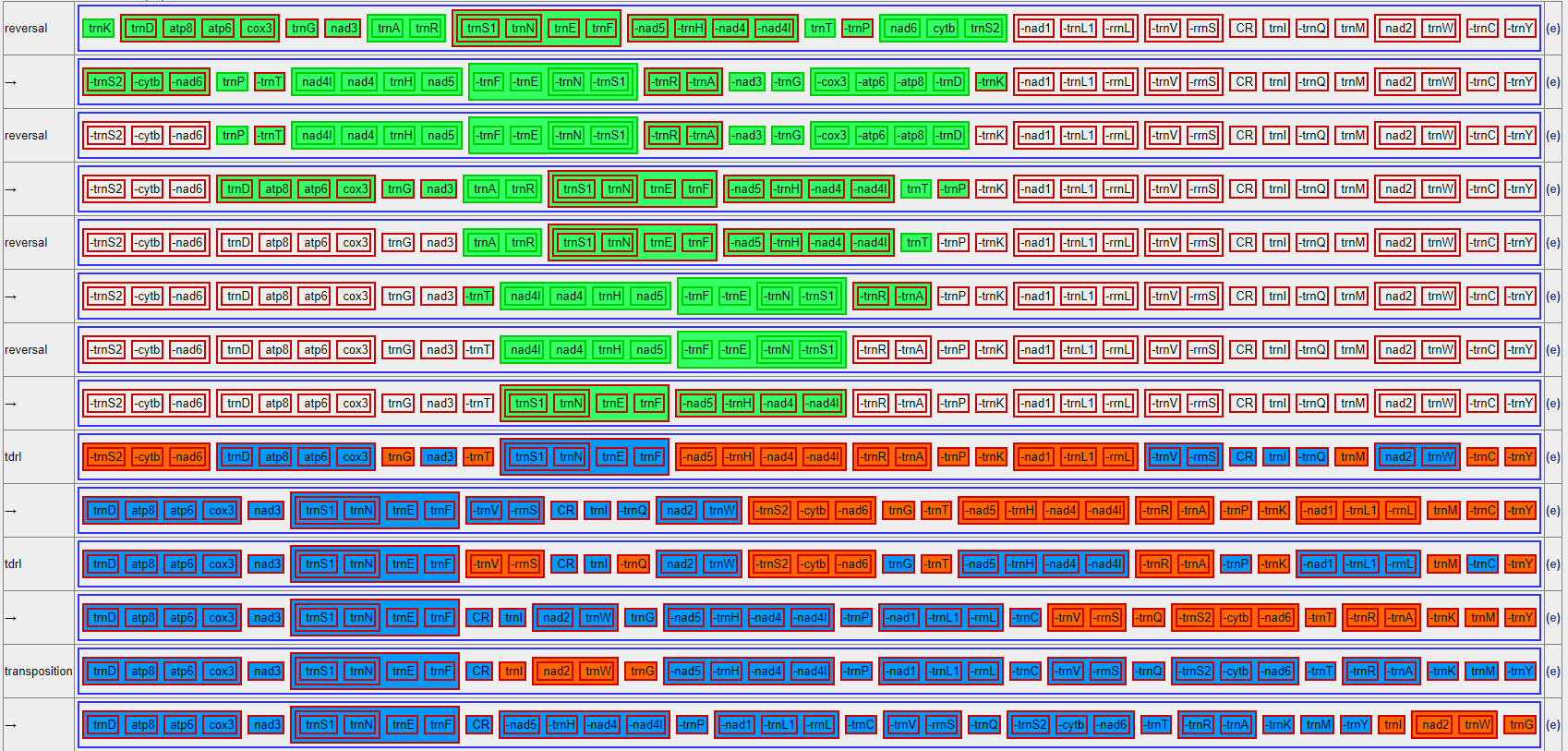


reversal


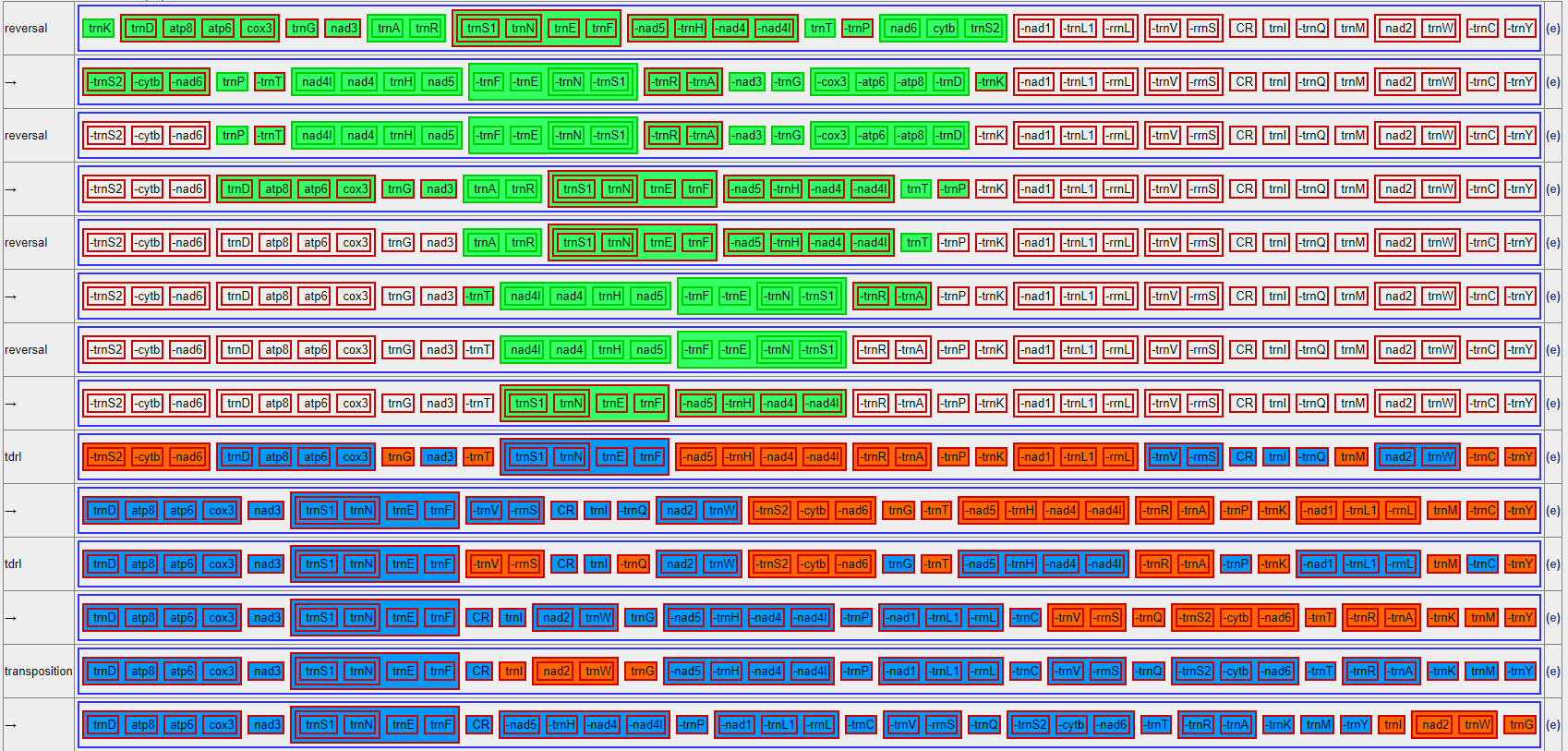


tdrl


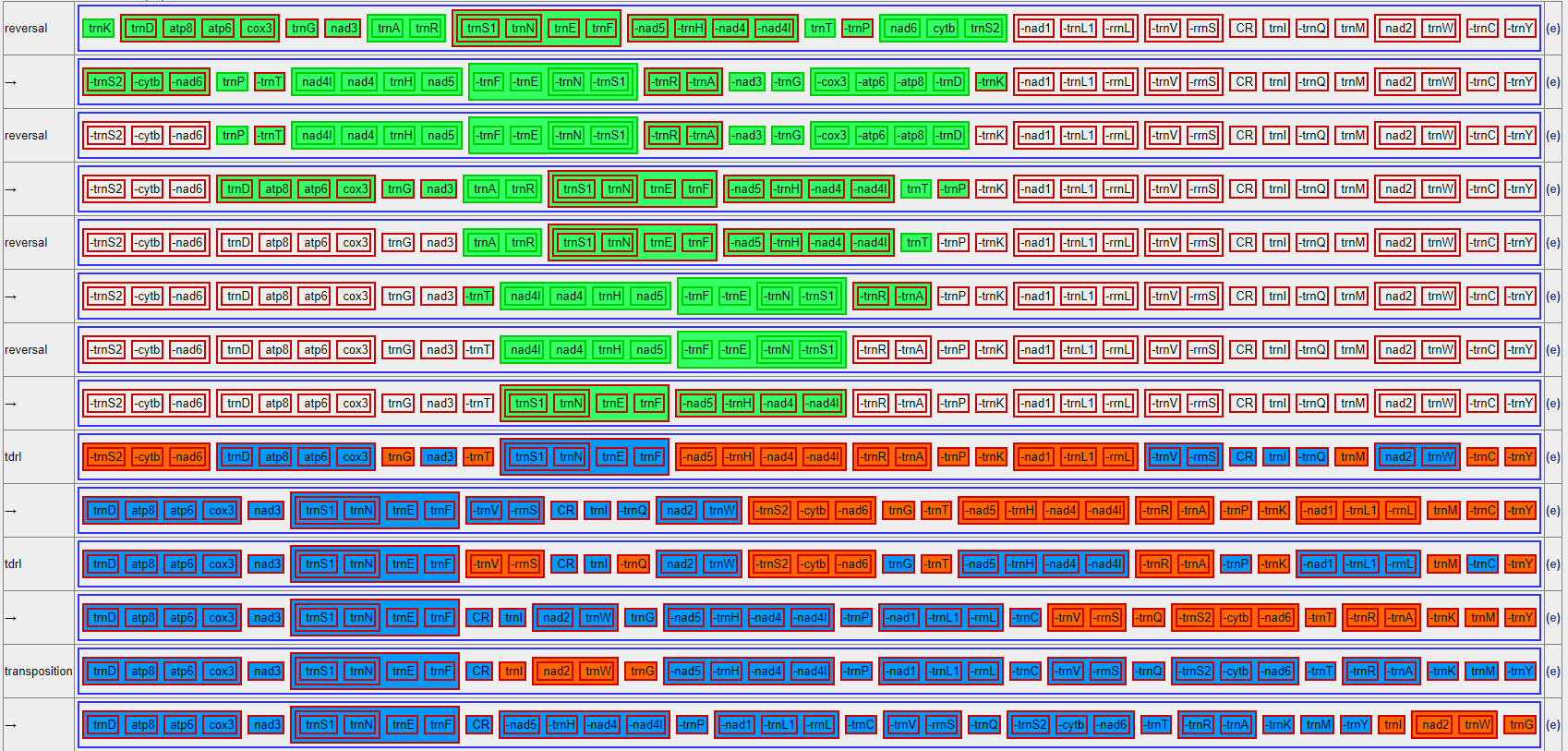


tdrl


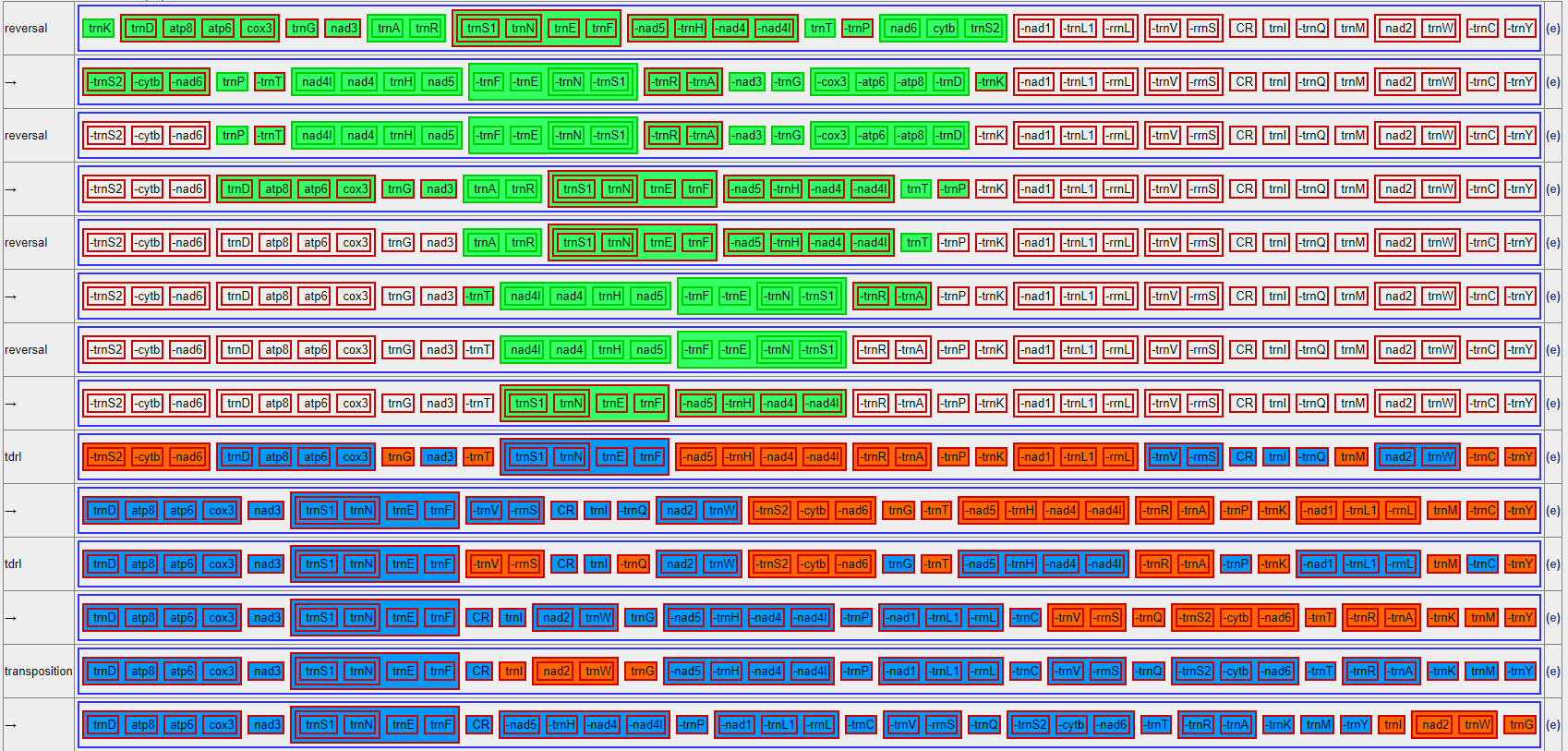


transposition


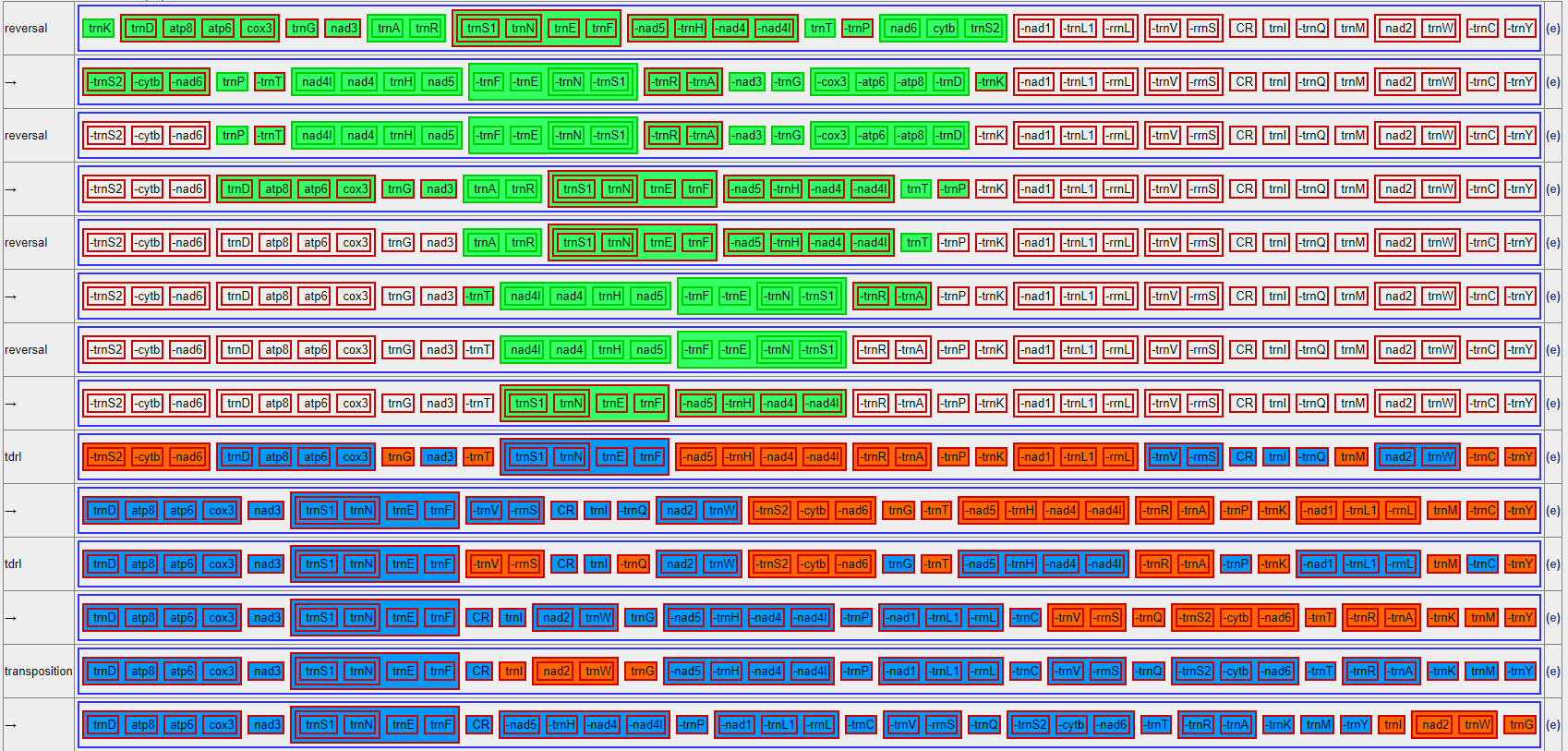


Supplementary figure 3. Overview of the mitochondrial rearrangement scenarios of **a** Eusirus cf. giganteus (G1 and G2) and **b** Charcotia amundseni as deduced by CREx (see discussion for details). Colours indicate which genes undergo rearrangements. The orange color indicates genes with tandem duplications and subsequent random gene loss (tdrl) and transpositions. Blue indicates genes without rearrangements, green indicates gene reversals.

**a**


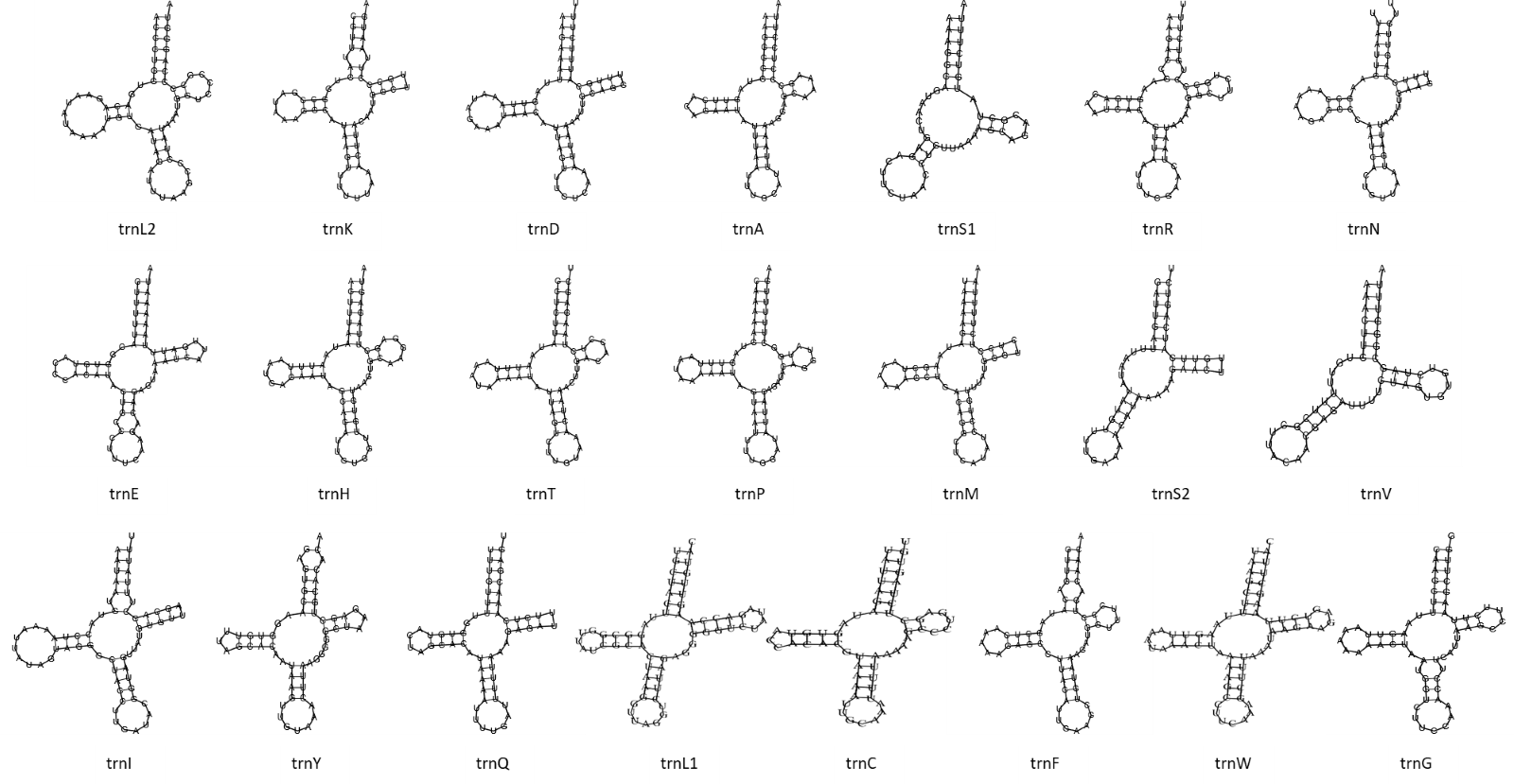


**b**


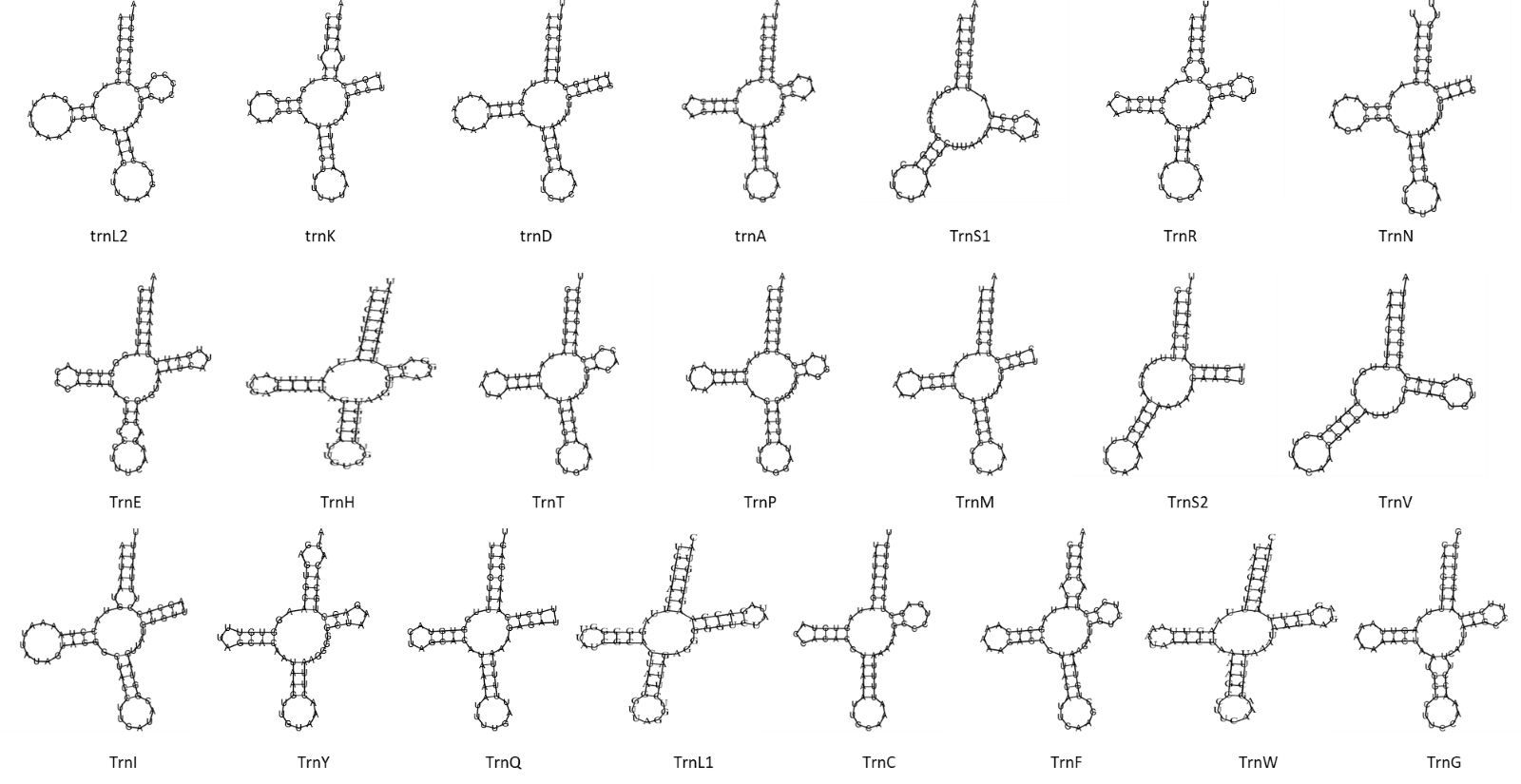


**c**


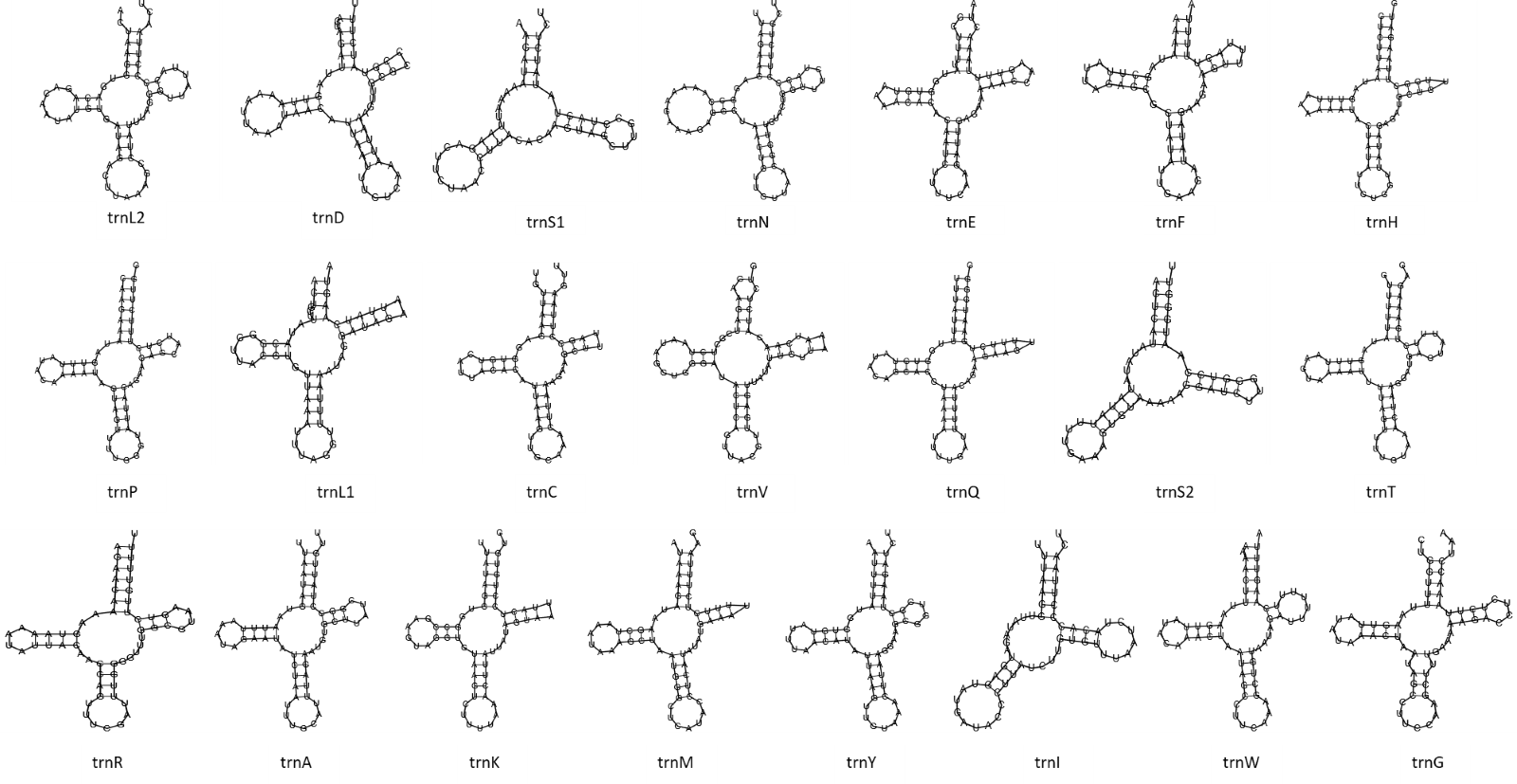


Supplementary figure 4. Putative secondary structures of the 22 tRNAs in **a** *Eusirus*cf. *giganteus* (G1) **b** *Eusirus* cf. *giganteus* (G2) **c** *Charcotia amundseni* mitogenomes.


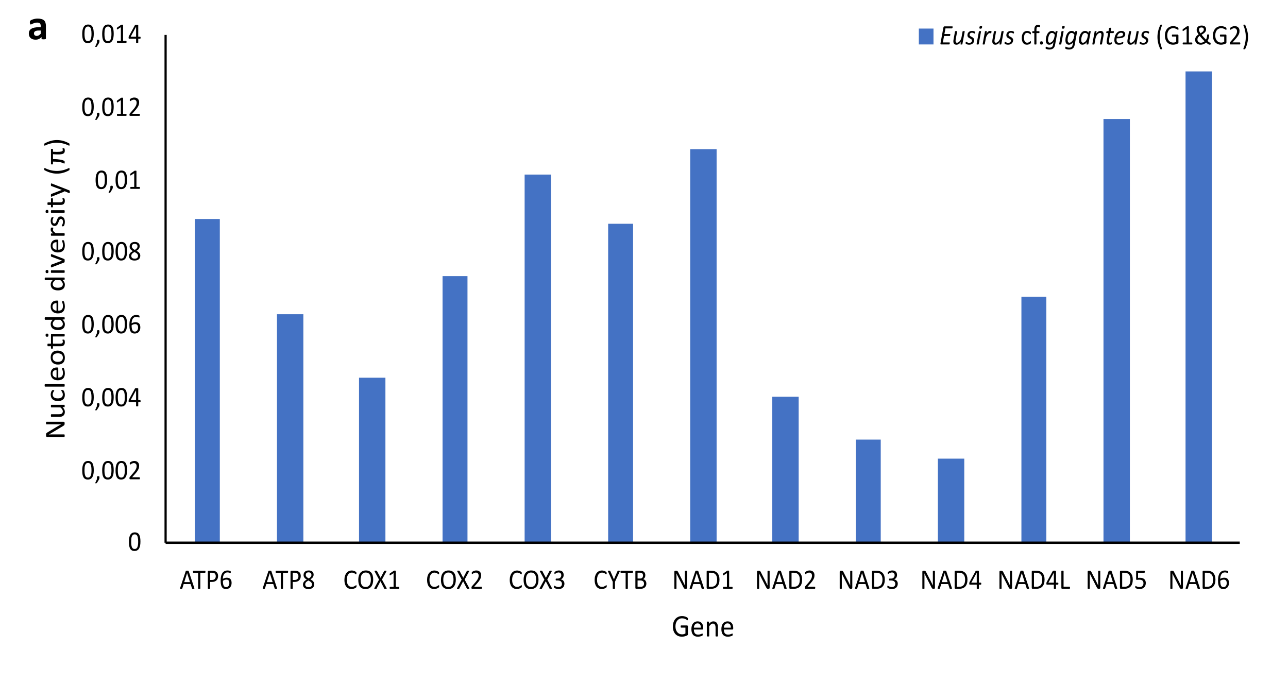

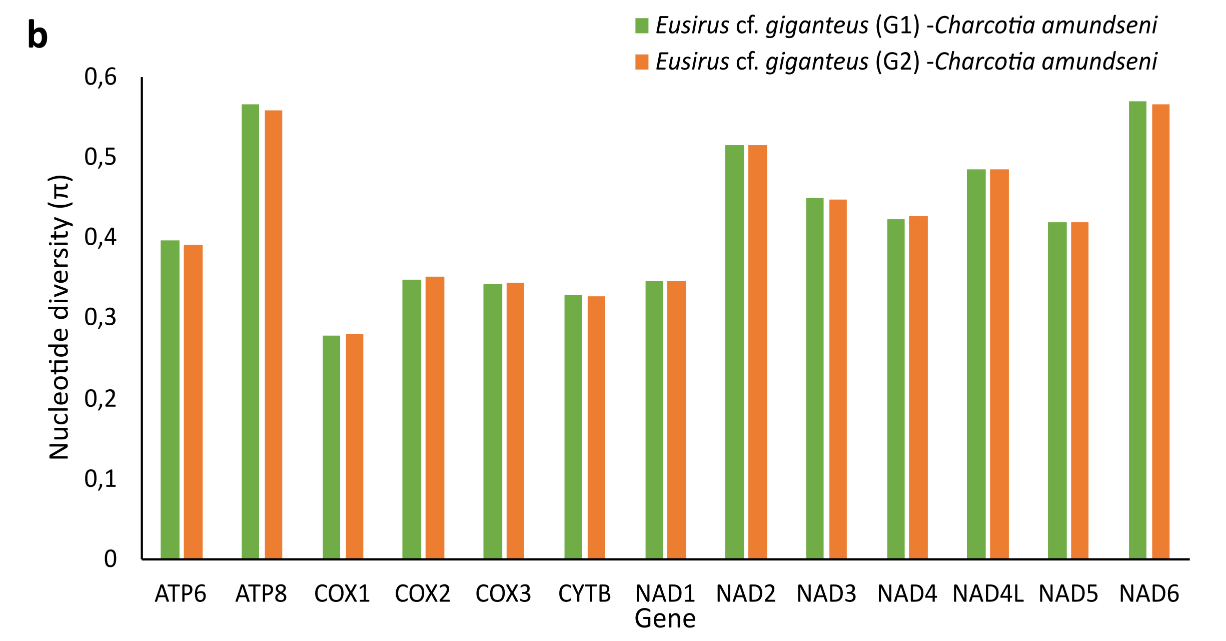


Supplementary figure 5. Nucleotide diversity $(\pi)$ for the mitochondrial protein coding genes estimated **a** between Eusirus cf. giganteus G1 and G2; **b** between Charcotia amundseni and Eusirus cf. giganteus G1 (green) and Charcotia amundseni and Eusirus cf. giganteus G2 (orange).
